# The Early Peopling of the Philippines based on mtDNA

**DOI:** 10.1038/s41598-020-61793-7

**Published:** 2020-03-17

**Authors:** Miguel Arenas, Amaya Gorostiza, Juan Miguel Baquero, Elena Campoy, Catarina Branco, Héctor Rangel-Villalobos, Antonio González-Martín

**Affiliations:** 1 Department of Biochemistry, Genetics and Immunology, University of Vigo, Vigo, Spain; 2 Biomedical Research Center (CINBIO), University of Vigo, Vigo, Spain; 3 Centre for Molecular Biology “Severo Ochoa”, Consejo Superior de Investigaciones Científicas (CSIC), Madrid, Spain; 4 Forensic Genetics and Identification Laboratory, GENOMICA S.A.U. Pharma Mar Group, Madrid, Spain; 5 Department of Biodiversity, Ecology and Evolution, University Complutense of Madrid, Madrid, Spain; 6 Institute of Research in Molecular Genetics, University of Guadalajara (CUCienega-UdeG), Ocotlán, Jalisco Mexico

**Keywords:** Evolutionary genetics, Population genetics, Population genetics

## Abstract

Despite the efforts made to reconstruct the history of modern humans, there are still poorly explored regions that are key for understanding the phylogeography of our species. One of them is the Philippines, which is crucial to unravel the colonization of Southeast Asia and Oceania but where little is known about when and how the first humans arrived. In order to shed light into this settlement, we collected samples from 157 individuals of the Philippines with the four grandparents belonging to the same region and mitochondrial variants older than 20,000 years. Next, we analyzed the hypervariable I mtDNA region by approximate Bayesian computation based on extensive spatially explicit computer simulations to select among several migration routes towards the Philippines and to estimate population genetic parameters of this colonization. We found that the colonization of the Philippines occurred more than 60,000 years ago, with long-distance dispersal and from both north and south migration routes. Our results also suggest an environmental scenario especially optimal for humans, with large carrying capacity and population growth, in comparison to other regions of Asia. In all, our study suggests a rapid expansion of modern humans towards the Philippines that could be associated with the establisment of maritime technologies and favorable environmental conditions.

## Introduction

Traditionally it is considered that the first humans who colonized Oceania departed from Southeast Asia (SEA) and reached the austral continent through Sumatra, Java and Timor^1^. According to this approach, the Philippine archipelago, constituted by more than 7,000 islands and located in a more northern region, would not be involved in the ancestral human migrations that colonized Oceania^2^. However, there are some studies that contradict this approach. The fossil record confirms that our modern humans were in the Philippines at least 40,000–50,000 years ago^3–5^, the genus *Homo* possibily 66,700 years ago^6,7^. Another finding is the presence in the archipelago of *Negritos* groups related to the first migrations of *Homo sapiens* outside Africa^4,8,9^. Indeed, several genetic studies detected an archaic substrate in current populations of the Philippines^4,5,10–14^. Hence, the human presence in the Philippines during the first stages of expansion of anatomically modern *Homo sapiens* is very likely. The recent finding of *Homo luzonensis*^7^ in the Philippines does not provide new information on the origin and expansion process of our species although it does demonstrate the importance of the archipelago to understand the past of our lineage. Consequently, the Philippines problably presented a relevant role in the out of Africa expansion of modern humans to colonize the SEA and Oceania.

Despite the relative certainty concerning the timing of the entry into the Philippines, only some studies investigated the population genetics and evolutionary processes occurring in the first expansion of modern humans towards this region. Alves, *et al*.^15^ and Pugach, *et al*.^16^ found that Paleolithic populations in Eurasia and Oceania, respectively, could have expanded with long-distance dispersal (LDD) events, rather than through a progressive expansion along the landscape. However, little is known about LDD and the migration routes used to reach the Philippines and about the Palaeolithic population dynamics in this region. The Philippines is an especially interesting region to test these population processes because its colonization required an expansion through large stretches of sea (bottlenecks), which could be done using boats or rafts as proposed for other regions of the world^17,18^.

In the present work, we investigated the timing of colonization, migration patterns and the population dynamics that occurred in the Philippine archipelago at the first stages of human expansion. Mitochondrial DNA (mtDNA) has been shown as a very informative genetic marker to study the genetic diversity and genetic relationships among populations of the Phillipines^19–23^. In agreement with these previous works and in order to address the previously presented questions, we collected sequences from the D-Loop region of mtDNA of 157 individuals selected from native volunteer donors from different geographic regions of the archipelago. All of them carried mitochondrial variants older than 20,000 years. It is important to consider that mtDNA is inherited to the maternal line (but see^24^), hence here we mainly make focus on the female evolutionary history of the archipelago, which is unequivocally related to the global history^25^. Next we applied approximate Bayesian computation (ABC) based on extensive spatially explicit computer simulations to identify the scenario of migration towards the archipelago that fits better with the data. We also estimated a variety of population genetic parameters informative about this settlement such as the time of the onset of the expansion, carrying capacity (available resources), demographics (including ancestral population size and population growth rate), mutation rate, migration rate, and proportion of migration events with LDD.

## Materials and Methods

### Sample collection and ethical statements

**Figure 1:**
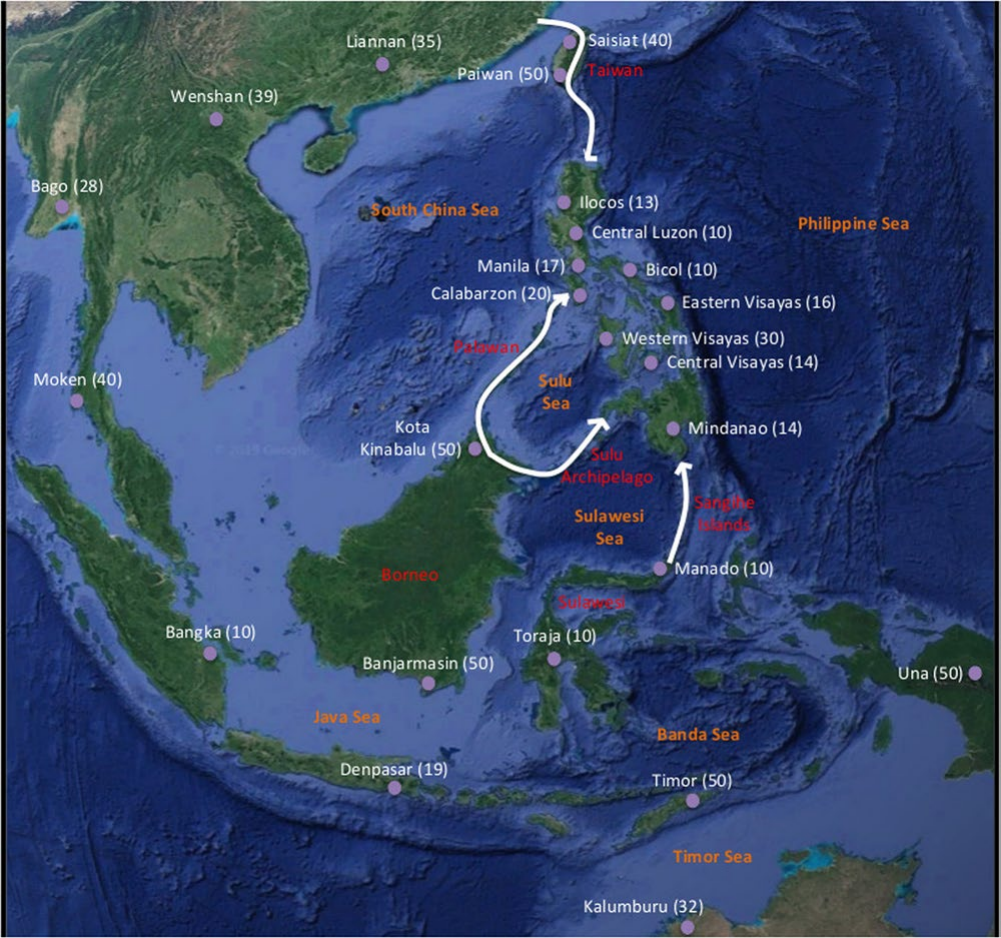
Studied landscape, samples and colonization routes. The figure shows the sampling locations applied in this study, including sample size (number of individuals) in parenthesis. The figure also shows four possible routes of early colonization of the Philippine archipelago: (1) northern route, from Taiwan through the Luzon Strait; (2) southwest route, from Borneo through Palawan; (3) central south route, from east of Borneo crossing the Sulu Archipelago towards western Mindanao; (4) southeast route, from Sulawesi through southern Mindanao through the Sangihe Islands. Combining these four routes we proposed seven possible colonization scenarios that are explored in this study: (*i*) migration allowing LDD events (*LDD*); (*ii*) All corridors (*AllCorr*), where the four routes are used; (*iii*) North corridor (*North*), colonization exclusively from the north; (*iv*) All south corridors (*AllSouth*); (*v*) Southwest corridor (*SW*); (*vi*) South Central corridor (*SC*); (*vii*) Southeast corridor (*SE*). The satellite image was obtained from the *Google Maps* application [Google Maps attribution: *Imagery (2019) Data SIO, NOAA, U.S. Navy, NGA, GEBCO, Landsat*/*Copernicus, Map data (2019) Google*; https://www.google.co.uk/maps/@10.0038529,108.7048144,3313765m/data=!3m1!1e3].

A total of 157 samples were collected covering most of the geographical area of the archipelago (Figs. 1 and S1; Table S1). The samples were obtained from Filipinos currently living in Spain but with known geographic origin in the Philippines. Each participant was informed about the study and signed an informed consent document for study participation according to the Helsinki protocol for the use of these samples. Indeed, the protocol was approved by the bioethics Committee of the Complutense University of Madrid. Next, the participants completed a survey about their origins and predecessors (including ethno-linguistic groups; Table S2). The samples were only collected from participants presenting the four grandparents in the region and accounting for the geographical diversity of the Philippines by donors from the three major regions of the archipelago: Luzon (n = 84), Visayas (n = 59) and Mindanao (n = 14). Moreover, given that the first two regions include a large number of islands, intraregional samples were collected. In particular, Luzón was divided into five subregions –Bicol (n = 10), Calabarzon (n = 20), Ilocos (n = 13), Manila (n = 17) and a central region (n = 24)– and, Visayas was divided into three subregions –West (n = 30), Center (n = 15) and East (n = 14)–.

### MtDNA amplification, sequencing and incorporation of additional sequences

Samples consisted of oral swabs (two per individual) that were kept dry in paper envelopes at 4 °C. Next, DNA was extracted by standard phenol-chloroform method including a final purification with AMICON filters following the instructions of the manufacturer (Merck). The mtDNA D-loop (control region) was sequenced for all 157 individuals using the BigDye Terminator kit v1.1 (Applied Biosystems), in two or three fragments depending on the samples between positions 16,000 and 600^26^. Amplified fragments were identified previously to the sequencing reaction in 4% agarose gel. Finally, the fragments were sequenced on Applied Biosystems ABI PRISM 3130 genetic analyzer. The genetic sequences were deposited at GenBank with accession numbers MH910797-MH910953.

The identification of spatial genetic variation is more accurate by considering a well-distributed sampling over the landscape^27^. Because of that, we included genetic samples from neighboring regions of the Philippines. Unfortunately, most of studies involving humans from those regions only shared sequences of the hypervariable I mtDNA region (HVR-I) and therefore posterior analyses were based on this region. We obtained from the bibliography genetic sequences from regions surrounding the Philippines (with a relatively even geographic distribution, Figs. 1 and S1; Table S1) and where each sampling location included genetic data from at least 10 individuals. We only considered sequences belonging to haplogroups older than 20,000 years^5^ to avoid lineages with origin posterior to the Philippines settlement^28^. The haplogroup assignation was performed with *HAPLOFIND*^29^ and *MitoTool*^30^ (Table S2). All the sequences were aligned with *MAFFT*^31^. The final multiple sequence alignment (MSA) included a total of 720 individuals collected from 25 different locations, from which 157 individuals of 9 populations of the Philippines were chosen and sequenced for this study. Details about sample size (number of individuals), geographic location and source (present study or other studies) are shown in Table S1 and depicted on the map in Figs. 1 and S1. Despite the limitations of our data due to the need of incorporating genetic information from different geographic areas, we found that our final dataset was sufficient to yield accurate model selection and estimation of population genetics parameters (see Results).

### Testing migration routes to the Philippines

Migration routes to the Philippines could affect the genetic diversity and population structure observed in this archipelago. Therefore, first, we investigated the fitting of diverse scenarios of migration towards the Philippines with the real data using ABC (details shown in following subsections).

Considering the geography of the Philippines, its neighboring regions and suggestions from previous studies^14,32,33^, we designed a total of 7 scenarios based on migration patterns and migration routes to the archipelago: (*i*) migration presenting LDD events (*LDD*, as suggested for Eurasia^15^; details about the LDD model are shown in the Supplementary Material) and, 6 scenarios of migration through different routes and without LDD proposed in the “*Beyer’s Wave Migration Theory*”^32^ and other studies^14,33^: (*ii*) a north route and three south routes (*AllCorr*), (*iii*) only a north route (*North*), (*iv*) only the three south routes (*AllSouth*), (*v*) only the southwest route (*SW*), (*vi*) only the south-central route (*SC*) and, (*vii*) only the southeast route (*SE*) (Figs. 1 and S2). Next, we evaluated non-nested scenarios by fitting with real data to identify the most likely scenario. In particular, in order to evaluate the influence of LDD, we evaluated: *LDD vs AllCorr* and, *LDD vs AllSouth vs North*. We also performed comparisons between other non-nested scenarios without LDD to explore the fitting of particular migration routes: *AllSouth vs North* (to explore migration from the north respect to migration from the south) and, *SW vs SC vs SE* (to explore the most likely migration route/s from the south).

### Spatially explicit computer simulations

We performed spatially explicit computer simulations with the evolutionary framework *SPLATCHE3*^34^. Information about the applied evolutionary models and landscape is presented in the Supplementary Material. The computer simulations were based on prior distributions for population genetic parameters following previous studies (Table S3). We assumed a range expansion with origin in Bangladesh at a time (*T*_*ANC*_) between 60,000 and 70,000 years ago^35^, with an ancestral population size (*N*_*ANC*_) between 5,000 and 50,000 individuals^36^. At each generation, individuals could migrate to adjacent demes at a migration rate (*MIG*_*R*_) between 0.2 and 0.3, following previous studies on Eurasia^15,37^. The population density for each deme was determined by the population growth rate (*N*_*GR*_) that varied between 0.4 and 1.0^15,37^ and the carrying capacity (*K*) that varied between 1,000 and 3,000^15,37^. For the LDD scenario, we applied a LDD proportion (*LDD*_*P*_) between 0.0001 (0.01%) and 0.05 (5%)^15,27^, we assumed migration distances sampled from a gamma distribution with parameters estimated from human data^38^ and we considered a maximum dispersal distance of 20 demes (500 km) to avoid unrealistically long LDD movements. Based on the previously simulated demographic history, we simulated genetic data for 720 individuals from 25 populations (Table S1 and Fig. 1). DNA sequence evolution was simulated according to a prior distribution for the mutation rate (*MUT*_*R*_) based on estimates of this rate in mtDNA presented in previous studies^39^ (Table S3). All these parameters were sampled from uniform prior distributions (Table S3) and were estimated with ABC under the best fitting evolutionary scenario.

### Approximate Bayesian computation for selection among evolutionary scenarios and parameters estimation

The real data was analyzed with the ABC approach^40^, which basically applies extensive computer simulations to estimate posterior probabilities of alternative scenarios and parameters by a statistical fitting between simulated and real data. Next, we describe the steps of our ABC method.

#### Computer simulations

For each of the seven evolutionary scenarios previously described, we performed a total of 100,000 spatially explicit computer simulations according to the prior distributions presented in Table S3. For each scenario, we also performed 100 additional and independent simulations (hereafter, *test datasets*) to evaluate the power of selection among evolutionary scenarios and parameters estimation (details shown later).

#### Summary statistics

Summary statistics (SS) from real and simulated datasets were obtained with *ARLEQUIN ver.3.5*^41^. To perform this task, the 25 populations were classified into five geographic groups (Fig. S1) that allowed the estimation of diverse group-based SS. Group 1 included samples from the north of the continent; group 2 included samples from the south of the continent; group 3 included samples from the Philippines; groups 4 and 5 included the remaining samples separated according to the Wallace line (Fig. S1). We considered that this general grouping was properly since the ABC performance reduces quickly as the number of statistics grows^40^. We applied a total of 18 SS with biological meaning: (*i*) genetic diversity in every group and in all groups together (pairwise differences, π; including standard deviation) and, (*ii*) genetic differentiation (F_ST_) between every two groups and all groups together. We found that this set of SS was informative for the ABC inferences (see validations in Results section). We did not apply partial least square (PLS) components to reduce the number of SS because our selected SS were informative enough and because PLS components can generate SS without biological meaning, being difficult to interpret and leading to biases when comparing components that are calculated separately^42^.

#### Selection among evolutionary scenarios

As indicated above, we generated 100,000 simulated datasets for each of the seven migration scenarios. In addition, we simulated 100 *test datasets* for each scenario to evaluate the accuracy of the estimation method. We applied three ABC approaches to perform the selection of the best-fitting evolutionary scenario with the real data: The rejection approach developed by Pritchard *et al*.^43^ (*Pr*) and, the rejection approach implemented in the *abc* package of *R*^44^ (*Rrej*) and the neuralnet (*Rnn*; nonlinear heteroscedastic regression^45^) approach also implemented in the *abc* package of *R*. Following the authors recommendation we retained 1% of simulations with SS closer to the SS of the real data^44^.

For the evaluation of the selection among alternative scenarios, we applied the 100 *test datasets* (considering them as true data) and also for *Rnn* the leave-one-out cross-validation method implemented in the *abc* library of R (*cv4postpr* function) that accounts for non-linearity adjustment based on 100 permutations and again with a tolerance of 1%^44^. The neuralnet method required more computational costs but, according to Csilléry *et al*.^44^, it could provide more accurate inferences.

Although traditionally the goodness of fit analysis is only applied to the preferred scenario, we performed goodness of fit analyses (based on principal component analysis (PCA) and a comparison among the null distributions from the selected SS^44^) to all the evolutionary scenarios designed in the study. In addtion, for these analyses of goodness of fit we included the position of the real dataset on the simulated parameters landscape for every comparison of scenarios, providing a preliminary view of the fitting of every scenario with the real data. Finally, we selected the scenario that better fits with the real data applying the ABC approaches previously described (*Pr*, *Rrej* and *Rnn*).

#### Parameters estimation

We performed the parameters estimation under the evolutionary scenario with LDD, which was the scenario that fitted best with the real data (see Results). The parameters estimation was performed with the multiple regression adjustment implemented in the program *ABCtoolBox*^46^. Following the software documentation, we retained the best 5,000 simulations (with SS closer to the SS of the real data) and also because they provided robust parameters estimation (see Results). The robustness of the parameter estimation was assessed with the 100 *test datasets* simulated under the *LDD* scenario, considering them as true data. For each *test dataset* and parameter we computed the distance between the true value and the estimated value (median, mean and mode).

## Results

We first describe the selection between alternative evolutionary scenarios of migration to the Philippines. After that, we present the parameters estimation under the best fitting evolutionary scenario.

### Migration to the Philippines occurred with long-distance dispersal and through all north and south migration routes

The power to select among the designed scenarios of migration to the Philippines varied depending on the scenarios that are evaluated. We obtained high posterior probabilities to identify the correct scenario under the different ABC methods when comparing (*i*) *LDD vs AllCorr* (probabilities around 1, Table S4A), (*ii*) *LDD vs AllSouth vs North* (probabilities generally above 0.8, Table S4B) and, (*iii*) *AllSouth vs North* (probabilities around 1, Table S4C). However, we obtained a lower accuracy when comparing the three scenarios with only migration routes from the south, *SW vs SC vs SE* (probabilities above 0.4, Table S4D). This result was expected because both SW and SC routes come from the same island (Borneo). In order to further explore this finding, we also evaluated the scenarios *SW vs SE* and there we found probabilities generally above 0.9 (Table S4E). Note that we performed comparisons among non-nested scenarios. If nested scenarios are analyzed together (i.e., comparing all the scenarios) the performance of the method will be poor because nested scenarios under certain evolutionary circunstances can lead to similar genetic signatures (i.e., *AllCorr vs AllSouth* could be similar in cases where under *AllCorr* the migration rate from the north is very small by chance sampling from the prior distribution).

Real data clearly fitted better with the scenario of LDD, with posterior probabilities above 0.9 under any ABC method (Table 1). Indeed, the analyses of goodness of fit also supported this finding (Fig. S4).

**Table 1:** Fitting of the studied evolutionary scenarios with the real data.

Evaluated evolutionary scenarios	Posterior probability		
	Pritchard’s method	R rejection method	R neuralnet method
*LDD*/*AllCorr*	**0.94**/0.06	**0.96**/0.04	**1.00**/0.00
*LDD*/*North*/*AllSouth*	**0.93**/0.05/0.02	**0.95**/1.30E-03/0.05	**1.00**/0.00/0.00
*North*/*AllSouth*	0.46/0.54	0.32/0.68	0.29/0.71
*SW*/*SC*/*SE*	0.35/0.33/0.31	0.39/0.38/0.23	0.54/0.24/0.22
*SW*/*SE*	0.52/0.48	0.59/0.41	0.69/0.31

Fitting of the studied evolutionary scenarios with real data based on the same comparisons (between non-nested scenarios) presented in the evaluation of the model selection (Table S4). The scenarios of migration to the Philippines are the following: LDD (LDD), all corridors without LDD (AllCorr), all the south corridors without LDD (AllSouth), only the north corridor without LDD (North), only the southwest corridor without LDD (SW), only the south-central corridor without LDD (SC), only the southeast corridor without LDD (SE). The table shows the probabilities based on the three applied ABC methods (see Materials and Methods) when fitting evolutionary scenario with the real data. Posterior probabilities above 0.9 (very high fitting) are shown in bold, note that the best fitting evolutionary scenario is LDD. The information of this table is graphically presented in the Figs. S3A–E.

Even considering that LDD was the best fitting evolutionary scenario, we explored the fitting of the different migration routes (without LDD) with the real data. We found that the migration to the Philippines through the south routes was generally more likely than the migration through the north route (Taiwan), but the north route could also be used (Table 1). When comparing the routes from the south, we found that all the routes were likely to be used, although the routes from Borneo (*SW* and *SC*) presented the highest probabilities. Note that the selection between *SW* and *SC* routes presented a poor performance (Table S4D), and therefore we cannot distinguish between *SW* and *SC* when fitting with the real data. The results slighly varied among ABC approaches.

### The settlement of the Philippines was rapid and caused by a rapidly increasing population

The estimation of the population genetic parameters was performed under the best-fitting evolutionary scenario (*LDD*). Concerning the validation of the parameters estimation, the method presented a performance with overall small estimation errors that always fell within the 50% HPDI with respect to the true value (Table S5). The estimated population genetic parameters for the real data are shown in Table 2. The precision was low for the estimation of some parameters, in particular for the time and population size at the onset of the expansion and the carrying capacity. This was expected because the estimation of these parameters requires considerable genetic signatures of ancestral processes, also found in other studies (e.g.^15,47^. However, we accounted for this uncertainty instead of fixing parameter values, since previous studies often disagree concerning these estimates^48^. We considered acceptable the precision of the estimation of the remaining parameters, presenting in general more narrow posterior distributions (Table 2).

**Table 2:** Population genetics parameters estimated with ABC under the best-fitting evolutionary scenario.

Parameter	Mode	Mean	Median	95% HPDI
Time of the onset of the expansion (*T*_*ANC*_)^a^	*60,151*	63,364	62,713	59,975–68,310
Population size at the onset of the expansion (*N*_*ANC*_)	*49,999*	46,316	47,060	25,760–49,886
Population growth rate (*N*_*GR*_)	0.822	*0.817*	0.825	0.591–0.998
Migration rate (*MIG*_*R*_)	0.285	*0.272*	0.280	0.212–0.299
Carrying capacity (*K*)	*3,000*	2,578	2,709	1,159–3,000
Mutation rate (*MUT*_*R*_)	*1.31E*^*−06*^	1.50E^−06^	1.41E^−06^	2.49E^−08^–3.08E^−06^
LDD proportion (*LDD*_*P*_)	0.040	*0.036*	0.038	0.011–0.049

For each parameter, we present the mode, mean and median of the estimated posterior distribution. In italic we highlight the most accurate measure (median, mean or mode) according to Table S5. 95% HPDI refers to the 95% highest posterior density interval.

^a^Time is shown in years and was estimated assuming a generation time of 25 years.

Our estimated date for the onset of the expansion of Anatomically Modern Humans (AMH) from Bangladesh (≈60 kya) fitted with previous studies^49^. The estimated ancestral size (above 30,000 individuals and probably near 50,000) was larger than we expected. However, some previous works (e.g.^50^,) also suggested large ancestral population sizes (near 40,000 individuals) in the region. Indeed, the estimated carrying capacity (≈3,000) and population growth rate per generation (≈0.82) were higher than expected, although they fell within the range estimated in other studies (i.e., in Europe and north of Africa the estimated carrying capacity varied between 500 and near 7,000^51^ and the estimated population growth rate in Eurasia varied between 0.4 and 0.9^15,51^). The estimated migration rate (≈0.27) was high but fell within estimates in Eurasia^15^. The proportion of LDD events (≈0.04) was in agreement with previous findings^15^. Although the estimed mutation rate (≈1.3E^−06^) fitted with other studies^52^, this parameter usually varies among studies, especially when they are obtained from different genetic markers (e.g.^39,52^).

## Discussion

Despite the efforts made to understand human phylogeography, there are still relevant regions of the world where the population genetics processes and migration patterns occuring in first colonizations have not been resolved. An especially interesting example is the Philippine archipelago. There is a clear evidence of an early settlement of the Philippines^6,8,10,11^, however little is known about the population genetics, migration routes and migration patterns that occurred in this settlement. Concerning its geography, the Philippines is a particularly relevant region to test the influence of the geography on human migration and to understand the causes of the currently observed population structure and genetic diversity.

Addressing the early settlement of the Philippines by the study of current genetic variants is complex given that its current composition is the result of a superposition of migratory waves distant in time^10^. Despite this complexity, here we investigated the fitting of diverse evolutionary scenarios of migration with real genetic data from a large number of individuals from the Philippines and neighboring regions in order to provide clues about the population genetics of the settlement and migration routes that could have occurred in this archipelago. Note that the Philippine archipelago, given its geographic location, could be colonized by two major routes: from the north (through Taiwan) and from the south (which could contemplate different routes from Borneo or from northern Sulawesi). Indeed, diverse combinations of potential migration routes could have occured. Moreover, this migration could also present LDD events. Here, we developed and applied an ABC method based on extensive spatially explicit computer simulations to determine whether the migration to the Philippines ocurred with LDD or by progressive (gradual) migration from neighboring regions through particular migration routes. Finally, we estimated demographic and evolutionary parameters under the best fitting evolutionary scenario.

We found that the evolutionary scenario fitting best with the real data was the one that presents migration with LDD. This finding supports a previous study on the Palaeolithic expansion on Eurasia^15^. Indeed, this result could be expected because the colonization of the archipelago implies displacements from island to island, which can lead to LDD events. The finding is also in agreement with some previous studies on the arrival of modern humans to Australia, which occurred crossing water barriers with watercrafts such as boats and rafts^17,18^. Next, we explored the fitting of particular migration routes with the real data to identify posible migratory pathways towards the Philippines. Our results showed that migration routes from both the north and the south were used to colonize the Philippines, although the probability was slighly higher for the colonization from the south. Moreover, within migration from the south, all the proposed routes were likely to occur. Altogether, we found that the most likely migratory scenario involving the early colonization of the Philippine archipelago by modern humans implied LDD events and migration through routes from both the north and the south. In theory, the southern route could be more used given the low sea level during the Last Glacial Maximum (LGM) period that led to the emergence of the continental mass of Sunda, connecting Indochina with the islands at the west of the Wallace line^53^. A northern route favored by the LGM seems more difficult since this would require the use of advanced navigation techniques to connect distant islands. However, for many authors navigation technologies were already developed by *Homo erectus*^54^ and with respect to our species, the presence of archaic fossil remains confirm that our ancestors must have navigated, more or less frequently, more than 60,000 years ago^1,17,18,55^. In this concern, our findings present genetic signatures of migration through LDD, which could fit with the use of techniques of navigation in the expansions of modern humans.

Concerning the demographic and environmental conditions under which the colonization of the archipelago was carried out, we found that the expansion from Bangladesh occurred at 60,000 years ago or earlier (HPDI around 60,000–68,000), a date consistent with findings from other disciplines regarding the human presence in the SEA and Australia (e.g.^1,56^,) and previous studies proposing the presence of our species in Australia at 65,000 years ago^57^. Next, the best fitting model (*LDD*) considers a rapid colonization of the Philippines (around 200 generations after the onset of the expansion, this is 5,000 years later) leading to an interval of 55,000–63,000 years ago. These dates agree with other studies (e.g.^4,5^,) and are slightly earlier than the dating of the remains of Callao Cave in Luzon (67,000 years old) although such remains have been considered as another species (*Homo luzonensis*)^6,7^. In any case it should be noted that for some authors these remains are fragmentary and taxonomically ambiguous^58^.

Our results described a rapid Palaeolithic expansion towards the Philippine archipelago. If that dispersal pattern is maintained for the posterior Neolithic expansion from current China^59^, which could be likely considering the technological and cultural innovations developed by Neolithic populations^59,60^ but still to be demonstrated in the study area, our results would support the variant of the *Out of Taiwan* theory (e.g.^22,59^,) known as the *Express Train to Polynesia*, that defends a rapid migration of Polynesian ancestors from the SEA towards the Southeast Pacific (e.g.^5^).

The estimated effective population size at the onset of the expansion varied between 25,000 and 50,000 individuals, with a higher probability for a large size. This large population size agrees with previous studies for the SEA^5,50,61^. Indeed, our estimates are based on mtDNA and thus they may represent only the female component of the population. For some authors the female demographic component can be about eight times higher than the male component^62–64^, although this was found for post-Neolithic periods characterized by patrilocal patterns. Studies focused on periods before the Neolithic, as in the present study, found a balanced sex-bias (e.g.^65^,) considering cultural, historical and geographical singularities of the study regions. We believe that this large population could favor the rapid spread of modern humans throughout the archipelago, in agreement with Jinam, *et al*.^5^. The estimated population growth rate and carrying capacity, directly related to *Annual Net Primary Production* (ANPP), fit with previously found population demographic patterns^66^, especially in hunter-gatherer societies^67^. The average value obtained for the population growth rate was 0.82 per generation (but ranging between 0.59 and 1.0). This result agrees with previous estimates that presented values between 0.3 and 0.9 (e.g.^15,51,68^,). On the other hand, the estimated carrying capacity (around 3,000) was higher than previous estimates in Eurasia (around 1,000 but with an upper estimate around 2,000)^15^ but lower than estimates for Neolithic populations (above 5,000)^51^. This suggests that Palaeolithic populations of the SEA could be favoured by an environment rich in natural resources, facilitating demographic growth. Actually, the paleoclimatic reconstructions of the SEA describe an ecological environment very similar to current sub-Saharan Africa and the presence of forests^69^, which could be beneficial for the living-style of Palaeolithic humans. Note that the SEA is one of the most important geographical spaces in our evolutionary history from where movements to Europe, America and Australia started^68^.

A possible consequence of the rapid demographic growth could be a rapid expansion throughout the islands of the Philippine archipelago. This affirmation is supported by the estimated high migration rates (around 27%) that reflect important human contingents movement, higher than those (23–25%) estimated for other groups of hunter-gatherers in Europe and north of Africa^51^. In addition, the estimated proportion of individuals migrating with LDD events was approximately 4%, similar to that obtained for Palaeolithic populations in Eurasia^15^, which again supports the rapid expansion of hunter-gatherers along Asia and expanding to Indonesia and Philippines.

The Philippines constitutes an important geographic region in the expansion of modern humans despite it was frecuently ignored in human evolutionary analyses of the SEA and Oceania. Here we explored the expansion of modern humans towards the Philippines and we found that this expansion was rapid, presenting LDD events, and following diverse migration routes that geography and existing technology could allow. We believe that the rapid expansion an explosive population growth could be favored by optimal environmental conditions present in the region at the time of the expansion.

## Supplementary information

Supplementary Material.

## Data Availability

The genetic sequences were deposited at GenBank with accession numbers MH910797-MH910953.

## References

[1] Malaspinas AS (2016). A genomic history of Aboriginal Australia. Nat..

[2] Nielsen R (2017). Tracing the peopling of the world through genomics. Nat..

[3] Barker G (2007). The ‘human revolution’ in lowland tropical Southeast Asia: the antiquity and behavior of anatomically modern humans at Niah Cave (Sarawak, Borneo). J. Hum. Evol..

[4] Jinam TA (2017). Discerning the Origins of the Negritos, First Sundaland People: Deep Divergence and Archaic Admixture. Genome Biol. Evol..

[5] Jinam TA (2012). Evolutionary history of continental southeast Asians: “early train” hypothesis based on genetic analysis of mitochondrial and autosomal DNA data. Mol. Biol. Evol..

[6] Mijares AS (2010). New evidence for a 67,000-year-old human presence at Callao Cave, Luzon, Philippines. J. Hum. Evol..

[7] Detroit F (2019). A new species of Homo from the Late Pleistocene of the Philippines. Nat..

[8] Aghakhanian F (2015). Unravelling the genetic history of Negritos and indigenous populations of Southeast Asia. Genome Biol. Evol..

[9] Reich D (2011). Denisova Admixture and the First Modern Human Dispersals into Southeast Asia and Oceania. Am. J. Hum. Genet..

[10] Lipson M (2014). Reconstructing Austronesian population history in Island Southeast Asia. Nat. Commun..

[11] Morseburg A (2016). Multi-layered population structure in Island Southeast Asians. Eur. J. Hum. Genet..

[12] Consortium HP-AS (2009). Mapping human genetic diversity in Asia. Sci..

[13] Mallick S (2016). The Simons Genome Diversity Project: 300 genomes from 142 diverse populations. Nat..

[14] Yew CW (2018). Genetic relatedness of indigenous ethnic groups in northern Borneo to neighboring populations from Southeast Asia, as inferred from genome-wide SNP data. Ann. Hum. Genet..

[15] Alves I (2016). Long-Distance Dispersal Shaped Patterns of Human Genetic Diversity in Eurasia. Mol. Biol. Evol..

[16] Pugach I (2018). The Gateway from Near into Remote Oceania: New Insights from Genome-Wide Data. Mol. Biol. Evol..

[17] Stringer C (2000). Palaeoanthropology. Coasting out of Africa. Nat..

[18] Balme J (2013). Of boats and string: The maritime colonisation of Australia. Quat. Int..

[19] Gunnarsdottir ED, Li M, Bauchet M, Finstermeier K, Stoneking M (2011). High-throughput sequencing of complete human mtDNA genomes from the Philippines. Genome Res..

[20] Loo JH (2011). Genetic affinities between the Yami tribe people of Orchid Island and the Philippine Islanders of the Batanes archipelago. BMC Genet..

[21] Delfin F (2014). Complete mtDNA genomes of Filipino ethnolinguistic groups: a melting pot of recent and ancient lineages in the Asia-Pacific region. Eur. J. Hum. Genet..

[22] Tabbada KA (2010). Philippine mitochondrial DNA diversity: a populated viaduct between Taiwan and Indonesia?. Mol. Biol. Evol..

[23] Soares P (2011). Ancient voyaging and Polynesian origins. Am. J. Hum. Genet..

[24] Luo S (2018). Biparental Inheritance of Mitochondrial DNA in Humans. Proc. Natl Acad. Sci. USA.

[25] Jorde LB (2000). The distribution of human genetic diversity: a comparison of mitochondrial, autosomal, and Y-chromosome data. Am. J. Hum. Genet..

[26] Anderson S (1981). Sequence and organization of the human mitochondrial genome. Nat..

[27] Mona S, Ray N, Arenas M, Excoffier L (2014). Genetic consequences of habitat fragmentation during a range expansion. Heredity.

[28] Behar DM (2012). A “Copernican” reassessment of the human mitochondrial DNA tree from its root. Am. J. Hum. Genet..

[29] Vianello D (2013). HAPLOFIND: a new method for high-throughput mtDNA haplogroup assignment. Hum. Mutat..

[30] Fan L, Yao YG (2011). MitoTool: a web server for the analysis and retrieval of human mitochondrial DNA sequence variations. Mitochondrion.

[31] Katoh K, Standley DM (2013). MAFFT multiple sequence alignment software version 7: improvements in performance and usability. Mol. Biol. Evol..

[32] Beyer, H. O. Earliest people of the Philippines. *Manila Bulletin* **27** (1950).

[33] Pawlik, A. F., Piper, P. J. & Mijares, A. S. B. In *Southern Asia, Australia and the Search for human origins* 135–147 (Cambridge University Press, 2014).

[34] Currat, M., Arenas, M., Quilodran, C. S., Excoffier, L. & Ray, N. SPLATCHE3: simulation of serial genetic data under spatially explicit evolutionary scenarios including long-distance dispersal. *Bioinformatics* **35**, 4480–4483 (2019). 10.1093/bioinformatics/btz311 PMC6821363 31077292

[35] Macaulay V (2005). Single, rapid coastal settlement of Asia revealed by analysis of complete mitochondrial genomes. Sci..

[36] Tenesa A (2007). Recent human effective population size estimated from linkage disequilibrium. Genome Res..

[37] Arenas M, Francois O, Currat M, Ray N, Excoffier L (2013). Influence of admixture and paleolithic range contractions on current European diversity gradients. Mol. Biol. Evol..

[38] Ray N, Excoffier L (2010). A first step towards inferring levels of long-distance dispersal during past expansions. Mol. Ecol. Resour..

[39] Soares P (2009). Correcting for purifying selection: an improved human mitochondrial molecular clock. Am. J. Hum. Genet..

[40] Beaumont MA (2010). Approximate Bayesian Computation in Evolution and Ecology. Annu. Rev. Ecol. Evol. Syst..

[41] Excoffier L, Lischer HEL (2010). Arlequin suite ver 3.5: a new series of programs to perform population genetics analyses under Linux and Windows. Mol. Ecol. Resour..

[42] Aeschbacher S, Beaumont MA, Futschik A (2012). A novel approach for choosing summary statistics in approximate Bayesian computation. Genet..

[43] Pritchard JK, Seielstad MT, Perez-Lezaun A, Feldman MW (1999). Population growth of human Y chromosomes: a study of Y chromosome microsatellites. Mol. Biol. Evol..

[44] Csillery K, Francois O, Blum MGB (2012). abc: an R package for approximate Bayesian computation (ABC). Methods Ecol. Evolution.

[45] Blum MGB, François O (2010). Non-linear regression models for Approximate Bayesian Computation. Stat. Comput..

[46] Wegmann D, Leuenberger C, Neuenschwander S, Excoffier L (2010). ABCtoolbox: a versatile toolkit for approximate Bayesian computations. BMC Bioinforma..

[47] Fagundes NJ (2007). Statistical evaluation of alternative models of human evolution. Proc. Natl Acad. Sci. USA.

[48] Benguigui M, Arenas M (2014). Spatial and temporal simulation of human evolution. Methods, frameworks and applications. Curr. Genomics.

[49] Stanyon R, Sazzini M, Luiselli D (2009). Timing the first human migration into eastern Asia. J. Biol..

[50] Li H, Durbin R (2011). Inference of human population history from individual whole-genome sequences. Nat..

[51] Pimenta J, Lopes AM, Comas D, Amorim A, Arenas M (2017). Evaluating the Neolithic Expansion at Both Shores of the Mediterranean Sea. Mol. Biol. Evol..

[52] Madrigal L (2012). High mitochondrial mutation rates estimated from deep-rooting Costa Rican pedigrees. Am. J. Phys. Anthropol..

[53] Allen, J., Golson, J. & Jones, R. *Sunda and Sahul: Prehistoric studies in Southeast Asia, Melanesia and Australia*. (Academic Press, 1977).

[54] von Koenigswald GHR (1958). Preliminary Report on a Newly-Discovered Stone Age Culture from Northern Luzon, Philippine Islands. Asian Perspect..

[55] Norman K (2018). An early colonisation pathway into northwest Australia 70-60,000 years ago. Quaternary Sci. Rev..

[56] Davidson I (2010). The Colonization of Australia and Its Adjacent Islands and the Evolution of Modern Cognition. Curr. Anthropol..

[57] Clarkson C (2017). Human occupation of northern Australia by 65,000 years ago. Nat..

[58] Reyes-Centeno H (2014). Genomic and cranial phenotype data support multiple modern human dispersals from Africa and a southern route into Asia. Proc. Natl Acad. Sci. USA.

[59] Diamond J, Bellwood P (2003). Farmers and their languages: the first expansions. Sci..

[60] Cavalli-Sforza LL, Menozzi P, Piazza A (1993). Demic expansions and human evolution. Sci..

[61] Atkinson QD, Gray RD, Drummond AJ (2008). mtDNA variation predicts population size in humans and reveals a major Southern Asian chapter in human prehistory. Mol. Biol. Evol..

[62] Hamilton G, Stoneking M, Excoffier L (2005). Molecular analysis reveals tighter social regulation of immigration in patrilocal populations than in matrilocal populations. Proc. Natl Acad. Sci. USA.

[63] Oota H, Settheetham-Ishida W, Tiwawech D, Ishida T, Stoneking M (2001). Human mtDNA and Y-chromosome variation is correlated with matrilocal versus patrilocal residence. Nat. Genet..

[64] Seielstad MT, Minch E, Cavalli-Sforza LL (1998). Genetic evidence for a higher female migration rate in humans. Nat. Genet..

[65] Wilkins JF, Marlowe FW (2006). Sex-biased migration in humans: what should we expect from genetic data?. Bioessays.

[66] Luck GW (2007). The relationships between net primary productivity, human population density and species conservation. J. Biogeogr..

[67] Binford, L. R. *Constructing Frames of Reference: An Analytical Method for Archaeological Theory Building Using Ethnographic and Environmental Data Sets*. (University of California Press, 2001).

[68] Eriksson A (2012). Late Pleistocene climate change and the global expansion of anatomically modern humans. Proc. Natl Acad. Sci. USA.

[69] Finlayson C (2005). Biogeography and evolution of the genus Homo. Trends Ecol. Evol..

