# Supplementary material for: The Early Peopling of the Philippines based on mtDNA
Source: Sci Rep. 2020 Mar 17;10:4901. doi: 10.1038/s41598-020-61793-7 (PMC7078265; doi:10.1038/s41598-020-61793-7)
Supplement: Supplementary file 1 — Supplementary Material. [file 41598_2020_61793_MOESM1_ESM.pdf]

Supplementary Material

The Early Peopling of the Philippines based on mtDNA

Miguel Arenas, Amaya Gorostiza, Juan Miguel Baquero, Elena Campoy, Catarina Branco, Hector Rangel-Villalobos and Antonio González-Martín

Spatially explicit computer simulations

For each of the seven evolutionary scenarios defined above, we simulated a Palaeolithic expansion with the evolutionary framework *SPLATCHE3*[1]. *SPLATCHE3* is a spatially explicit computer simulator widely used to investigate human evolution[2-8]. This program implements a forward-in-time simulation of the entire population under demographic parameters, such as population size at the onset of the expansion and population growth rate, during a given number of generations. This simulation is performed over a two-dimensional (2D) landscape (grid of demes) that also considers parameters such as carrying capacity per deme (number of individuals that can be sustained by the resources of the deme) and migration rate per deme (individuals can migrate to neighboring demes under a 2D stepping-stone migration model[9]). The landscape used for this study was obtained by a geographic information system (GIS) and includes 44,064 demes with deme size 25 × 25 km (Figures S1 and S2) that covers the geography from north of Australia (south of the landscape) to Bangladesh (northwest) and Taiwan (northeast). We artificially added land bridges connecting some nearby islands (Borneo-Malaysia, Borneo-Sumatra, Borneo-Jakarta, Jakarta-Timor, Timor-north of Australia, Molucas-New Guinea) and by including routes of migration to the Philippines (Luzon-Taiwan (North route), Borneo-Palawan-Mindoro (SW route), Borneo-Sulu-Mindanao (SC route) and Sulawesi-Mindanao (SE route); see Figures 1 and S2) to allow the settlement of such islands in scenarios ignoring long-distance dispersal (LDD). These bridges were specified with half of the carrying capacity assigned to land regions to mimic corridors with little resources, in agreement with[6]. For the LDD scenario, we applied the LDD model developed by Ray and Excoffier[10] that allows individuals to migrate to any (empty or colonized) deme. In this model the

direction of LDD movements is randomly selected and the length of LDD movements is modeled with a gamma distribution that can be truncated to fix a maximum distance of movement[10]. After the demographic simulation, *SPLATCHE3* performs a backward-in-time (coalescent) simulation that generates the evolutionary history of a user-specified sample accounting for the history of the whole population stored in the previous step[11]. Next, the program simulates the genetic data of the sample. To do so, a randomly selected sequence is assigned to the root node (most recent common ancestor, MRCA) of the previously simulated coalescent history and that sequence is evolved forward-in-time over the coalescent history, introducing mutation events along branches according to the specified mutation rate and a model of molecular evolution, to finally generate genetic sequences for every node of the sample[12,13]. Note that the simulated genetic data is influenced by the specified population genetics parameters (i.e., a larger population size can lead to a higher genetic diversity)[14]. Note that the computer simulations required assumptions due to the complexity of the studied scenarios and to avoid overparameterization of the models. For example, the applied models assumed a homogeneous geographic distribution of environmental conditions (i.e., carrying capacity) and population genetic processes (i.e., population growth).

Supplementary Tables

Table S1. Samples considered for selection among evolutionary scenarios and parameters estimation. The distribution of these samples on the landscape is shown in Figures 1 and S1.

Sample	Country	Sample size	Reference
Wenshan	China	39	[15]
Liannan	China	35	[15]
Saisiat	China (Taiwan)	40	[16]
Paiwan	China (Taiwan)	50	[17]
Bago	Myanmar	28	[18]
Moken	Thailand	40	[19]
Cham	Vietnam	50	[20]
Bicol	Philippines	10	This study
Calabarzon	Philippines	20	This study
Ilocos	Philippines	13	This study
Manila	Philippines	17	This study
Luzon Central	Philippines	24	This study
Visayas West	Philippines	30	This study
Visayas Central	Philippines	14	This study
Visayas East	Philippines	15	This study
Mindanao	Philippines	14	This study
Kota Kinabalu	Malaysia (Borneo)	50	[21]
Banjarmasin	Indonesia (Borneo)	50	[22]
Bangka	Indonesia (Sumatra)	10	[23]
Kalumburu	Australia	32	[24]
Denpasar	Indonesia (Bali)	19	[21]
Una	Indonesia (Papua)	50	[25]
Manado	Indonesia (Sulawesi)	10	[21]
Toraja	Indonesia (Sulawesi)	10	[21]
Timor	Timor-Leste	50	[26]
**TOTAL**	25	720	

Table S2. Haplogroups identified for the sequences provided in the present study. For the Philippine individuals (*entries*) included in the present study the table shows region, subregion, ethno-linguistic group and specific haplogroup. Details about the identification of haplogroups are shown in Methods.

Entry	Philippines region	Philippines subregion	Ethno-linguistic groups	Haplogroup/s
1	Luzon	Bicol	Tagalog, Chavacano	B4a1a3a
2	Luzon	Bicol	Tagalog, Bikol	M7b1a2a
3	Luzon	Bicol	Tagalog, Waray, Bikol	B5b1c
4	Luzon	Bicol	Tagalog, Bikol	B5b1c
5	Luzon	Bicol	Tagalog, Pampango	B4c1b2a2
6	Luzon	Bicol	Tagalog, Bikol	B4c1b2a2
7	Luzon	Bicol	Tagalog	B5b1c
8	Luzon	Bicol	Tagalog	B4c1b2a2
9	Luzon	Bicol	Waray	M7c1a4a; M7c1c3
10	Luzon	Bicol	Tagalog	M7b1a1i
1	Luzon	Calabarzon	Tagalog	M7c1c3a
2	Luzon	Calabarzon	Tagalog	F1a
3	Luzon	Calabarzon	Tagalog	F3b1
4	Luzon	Calabarzon	Tagalog	M7b1a2a
5	Luzon	Calabarzon	Tagalog, Waray	M7c1a4a; M7c1c3
6	Luzon	Calabarzon	Tagalog	B4c1b2a2
7	Luzon	Calabarzon	Tagalog	M7b1a2a
8	Luzon	Calabarzon	Tagalog	M7c1a4a; M7c1c3
9	Luzon	Calabarzon	Tagalog	F1a1a

Entry	Philippines region	Philippines subregion	Ethno-linguistic groups	Haplogroup/s
1	Luzon	Manila	Tagalog	B4b1a2
2	Luzon	Manila	Tagalog	B4c1b2
3	Luzon	Manila	Tagalog	M7c1a4a; M7c1c3
4	Luzon	Manila	Tagalog, Cebuano	M7c1c3a
5	Luzon	Manila	Tagalog	B4b1a2
6	Luzon	Manila	Tagalog	B4b1a2
7	Luzon	Manila	Tagalog	M7c1a4a; M7c1c3
8	Luzon	Manila	Tagalog	B4a1a; B4a1c4
9	Luzon	Manila	Tagalog	B4b1a2c
10	Luzon	Manila	Tagalog	B4a1a; B4a1c4
11	Luzon	Manila	Tagalog	F1a3a
12	Luzon	Manila	Tagalog	M7b1a1; M7b1a1i
13	Luzon	Manila	Tagalog, Hiligaynon, Chavacano	M7c1a4a; M7c1c3; D4s
14	Luzon	Manila	Tagalog	M7b1a2a
15	Luzon	Manila	Tagalog	B5b1c
16	Luzon	Manila	Tagalog	F1a
17	Luzon	Manila	Tagalog	F1a3a
1	Visayas	Visaya Central	Tagalog, Waray	B4b1a2
2	Visayas	Visaya Central	Tagalog	B4c1b2a2

10	Luzon	Calabarzon	Tagalog	M7c1a4a; M7c1c3
11	Luzon	Calabarzon	Tagalog, Chavacano	F1a3a
12	Luzon	Calabarzon	Tagalog, Ilocano	F4b
13	Luzon	Calabarzon	Tagalog, Cebuano, Warai,	B4a1c3
14	Luzon	Calabarzon	Tagalog	B4a1a; B4a1c4
15	Luzon	Calabarzon	Tagalog	B4b1a2
16	Luzon	Calabarzon	Tagalog	B4c1b2a2
17	Luzon	Calabarzon	Tagalog	B4a1a; B4a1c4
18	Luzon	Calabarzon	Tagalog	B4a1a; B4a1c4
19	Luzon	Calabarzon	Tagalog, Cebuano, Bikol, Chavacano	F1a
20	Luzon	Calabarzon	Tagalog, Bikol	M7c1c3e
1	Luzon	Ilocos	Tagalog, Ilocano	F1a4a1
2	Luzon	Ilocos	Tagalog, Pangasinan,	F1a4a1
3	Luzon	Ilocos	Tagalog, Ilocano	B4b1a2
4	Luzon	Ilocos	Tagalog	M7c1a4a; M7c1c3
5	Luzon	Ilocos	Tagalog, Ilocano,	M7c1a4a; M7c1c3
6	Luzon	Ilocos	Tagalog, Ilocano,	M7c1a4a; M7c1c3
7	Luzon	Ilocos	Tagalog, Ilocano, Pangasinan	F3b1
3	Visayas	Visaya Central	Tagalog	M5; M7b1; M13b
4	Visayas	Visaya Central	Tagalog	M75
5	Visayas	Visaya Central	Tagalog, Cebuano	R24a
6	Visayas	Visaya Central	Tagalog, Cebuano, Bikol	F1a4a1
7	Visayas	Visaya Central	Tagalog, Cebuano, Hiligaynon	M7c; M50
8	Visayas	Visaya Central	Tagalog	B4a1a; B4a1c4
9	Visayas	Visaya Central	Tagalog	B4a1a; B4a1c4
10	Visayas	Visaya Central	Tagalog, Cebuano, Hiligaynon	B5b1c
11	Visayas	Visaya Central	Tagalog, Cebuano, Hiligaynon,	D6c
12	Visayas	Visaya Central	Tagalog, Chavacano	B4c1b2a2
13	Visayas	Visaya Central	Tagalog, Cebuano	B4b1a2b
14	Visayas	Visaya Central	Tagalog	M7c1a4a; M7c1c3; D4j6; D4s
1	Visayas	Visaya Western	Tagalog, Hiligaynon	F1a3a
2	Visayas	Visaya Western	Tagalog	M7b1a2a
3	Visayas	Visaya Western	Tagalog, Hiligaynon	B4b1a2b
4	Visayas	Visaya Western	Tagalog, Bikol	M7b1a1a; M7b1a1d;
5	Visayas	Visaya Western	Tagalog, Cebuano, Hiligaynon	M7b1a1i
6	Visayas	Visaya Western	Tagalog, Ilonggo	M7c1a4a; M7c1c3
7	Visayas	Visaya Western	Tagalog, Aklanon	M7c1a4a; M7c1c3
8	Visayas	Visaya Western	Tagalog, Hiligaynon	B5b1c

8	Luzon	Ilocos	Tagalog, Pangasinan,	B4b1a2
9	Luzon	Ilocos	Tagalog	M7c; E1a1a
10	Luzon	Ilocos	Tagalog	B5b1c
11	Luzon	Ilocos	Tagalog	F1a3a
12	Luzon	Ilocos	Tagalog, Cebuano, Hiligaynon, Chavacano	B5b1c
13	Luzon	Ilocos	Tagalog	B4c1b2a2
1	Luzon	Luzon Central	Tagalog	B4b1a2
2	Luzon	Luzon Central	Tagalog	R9c1a
3	Luzon	Luzon Central	Tagalog	B4a1a; B4a1c4
4	Luzon	Luzon Central	Tagalog	B4a1a; B4a1c4
5	Luzon	Luzon Central	Tagalog, Bikol	B4a1a; B4a1c4
6	Luzon	Luzon Central	Tagalog, Pampango	M7c1a4a; M7c1c3
7	Luzon	Luzon Central	Tagalog	B4a1a; B4a1c4
8	Luzon	Luzon Central	Tagalog	B4b1a2
9	Luzon	Luzon Central	Tagalog	B4b1a2
10	Luzon	Luzon Central	Tagalog, Ilocano, Hiligaynon	M7b1a1d; M7b1a1e
11	Luzon	Luzon Central	Tagalog, Hiligaynon	B4c1b2a2
12	Luzon	Luzon Central	Tagalog, Pampango	M7c1a4a; M7c1c3
13	Luzon	Luzon Central	Tagalog, Waray	M7b1a1i
14	Luzon	Luzon Central	Tagalog, Cebuano	B4a1a; B4a1c4
15	Luzon	Luzon Central	Tagalog	M7c1a4a; M7c1c3
16	Luzon	Luzon Central	Tagalog, Ilocano	R9c1
17	Luzon	Luzon Central	Tagalog	M7c1a4a; M7c1c3
9	Visayas	Visaya Western	Tagalog, Cebuano	B4b1a2b
10	Visayas	Visaya Western	Tagalog	B4b1a2b
11	Visayas	Visaya Western	Tagalog, Bikol	R9c1a
12	Visayas	Visaya Western	Tagalog, Pampango	B4b1a2b
13	Visayas	Visaya Western	Tagalog, Hiligaynon, Chavacano	M7b1a2a
14	Visayas	Visaya Western	Tagalog, Hiligaynon, Cebuano	B4a1a; B4a1c4
15	Visayas	Visaya Western	Tagalog	M7c; M50; M62'68
16	Visayas	Visaya Western	Tagalog, Hiligaynon	B4a1a3a
17	Visayas	Visaya Western	Tagalog, Hiligaynon	R9c1a
18	Visayas	Visaya Western	Tagalog, Hiligaynon, Pampango	M7c1a4a; M7c1c3
19	Visayas	Visaya Western	Tagalog	B4b1a2b
20	Visayas	Visaya Western	Tagalog	M7c1a4a; M7c1c3
21	Visayas	Visaya Western	Tagalog	B5b1c
22	Visayas	Visaya Western	Tagalog, Waray, Ilocano	M7c1a4a; M7c1c3
23	Visayas	Visaya Western	Tagalog, Chavacano	M7c1a4a; M7c1c3
24	Visayas	Visaya Western	Tagalog	F1a3a
25	Visayas	Visaya Western	Tagalog, Cebuano	F1a3a
26	Visayas	Visaya Western	Tagalog, Chavacano	R9c1a
27	Visayas	Visaya Western	Tagalog, Bikol, Hiligaynon	M7b1a1i
28	Visayas	Visaya Western	Tagalog, Bicolano	B4a1a; B4a1c4

18	Luzon	Luzon Central	Tagalog, Bicolano	B4b1a2
19	Luzon	Luzon Central	Tagalog	R9c1a
20	Luzon	Luzon Central	Tagalog, Hiligaynon	F1a1d
21	Luzon	Luzon Central	Tagalog, Pangasinan	F1a3a
22	Luzon	Luzon Central	Tagalog	R9b1a1
23	Luzon	Luzon Central	Tagalog	B4b1a2
24	Luzon	Luzon Central	Tagalog, Hiligaynon	B5b1c
1	Mindanao	Caraga	Tagalog	B4a1a; B4a1c4
2	Mindanao	Caraga	Tagalog, Cebuano, Surigaonon	B4b1a2b
3	Mindanao	Mindanao	Tagalog	M7c1a4a; M7c1c3
4	Mindanao	North of Mindanao	Tagalog, Cebuano, Cahavacano	B4b1a2
5	Mindanao	North of Mindanao	Tagalog, Cebuano	M7c1a4a; M7c1c3
6	Mindanao	North of Mindanao	Tagalog	P10
7	Mindanao	Mindanao Muslim	Tagalog, Cebuano, Maranao	D6a2
8	Mindanao	Mindanao Muslim	Tagalog, Pampango	M73a
9	Mindanao	Zamboanga	Tagalog, Cebuano	B4b1a2
10	Mindanao	Zamboanga	Tagalog, Waray	F1a4a1
11	Mindanao	Zamboanga	Tagalog	R9c1
12	Mindanao	Zamboanga	Tagalog	N22
13	Mindanao	Davao	Tagalog, Chavacano	B4a1a; B4a1c4
14	Mindanao	Soccsksargen	Tagalog, Waray	M7c1a4a; M7c1c3
29	Visayas	Visaya Western	Tagalog, Hiligaynon, Cuyonon, Agutaynen	F3b1
30	Visayas	Visaya Western	Tagalog, Hiligaynon, Chavacano	R9c1a
1	Visayas	Visaya Eastern	Tagalog, Ilocano	B4b1a2
2	Visayas	Visaya Eastern	Tagalog	F3b1a
3	Visayas	Visaya Eastern	Tagalog, Waray	M7b1a1i
4	Visayas	Visaya Eastern	Tagalog, Cebuano, Waray, Boholano	M45
5	Visayas	Visaya Eastern	Tagalog	M7c1a4a; M7c1c3
6	Visayas	Visaya Eastern	Tagalog	M7c1a4a; M7c1c3
7	Visayas	Visaya Eastern	Tagalog	B4b1a2
8	Visayas	Visaya Eastern	Tagalog, Ilocano	B4a1a; B4a1c4
9	Visayas	Visaya Eastern	Tagalog	B4b1a2
10	Visayas	Visaya Eastern	Tagalog, Cebuano, Hiligaynon	R9c1a
11	Visayas	Visaya Eastern	Tagalog, Bicolano	B4b1a2
12	Visayas	Visaya Eastern	Tagalog., Hiligaynon	B5b1c
13	Visayas	Visaya Eastern	Tagalog	B4b1a2
14	Visayas	Visaya Eastern	Tagalog	B4; B4b1
15	Visayas	Visaya Eastern	Tagalog	B4a1a; B4a1c4

Table S3. Prior distributions for the population genetic parameters of the studied evolutionary scenarios.

Parameter	Distribution	Source references	Model
Time of the onset of the expansion (T_{ANC})[1]	Uniform (60,000–70,000)	[23,27]	All models
Population size at the onset of the expansion (N_{ANC})	Uniform (5,000–50,000)	[28]	All models
Population growth rate (N_{GR})	Uniform (0.4–1.0)	[2]	All models
Migration rate (MIG_R)	Uniform (0.2–0.3)	[2]	All models
Carrying capacity (K)	Uniform (100–3,000)	[2]	All models
Mutation rate (MUT_R)	Uniform ($1E^{-15}$–$1E^{-05}$)	[29-31]	All models
LDD proportion (LDD_P)	Uniform ($1E^{-4}$–0.05)	[2]	*LDD*

[1]Time is shown in years.

Tables S4. Power of the ABC methods for selecting among non-nested evolutionary scenarios. The selection among evolutionary scenarios was performed with three different ABC approaches: (*i*) the Pritchard's approach[32] (*Pr*), through 100 *test datasets* and tolerance 0.1%; (*ii*) the leave-one-out cross-validation approach implemented in the *abc* library of R (R; function *cv4postpr*)[33] through 100 random simulations under the rejection approach (*Rrej*)[34] and, (*iii*) the neuralnet approach (*Rnn*) that accounts for non-linearity adjustment and with tolerance 1% according to the author's recommendation[33]. The tables show the probabilities of the three approaches when fitting the studied combinations of non-nested evolutionary scenarios to the true data. The identification of the correct scenario is shown in bold.

Table S4A. Power to distinguish among *LDD vs AllCorr*.

	LDD	*AllCorr*
	Pr Rrej Rnn	*Pr Rrej Rnn*
LDD	**0.99 0.98 0.98**	0.00 0.01 0.02
AllCorr	0.01 0.02 0.02	**1.00 0.99 0.98**

Table S4B. Power to distinguish among *LDD vs AllSouth vs North*.

	LDD	*AllSouth*	*North*
	Pr Rrej Rnn	*Pr Rrej Rnn*	*Pr Rrej Rnn*
LDD	**0.64 0.83 0.72**	0.05 0.17 0.23	0.00 0.03 0.05
AllSouth	0.36 0.12 0.23	**0.95 0.82 0.76**	0.00 0.01 0.01
North	0.00 0.05 0.05	0.00 0.01 0.01	**1.00 0.96 0.94**

Table S4C. Power to distinguish among *AllSouth vs North*.

	AllSouth	*North*
	Pr Rrej Rnn	*Pr Rrej Rnn*
AllSouth	**1.00 0.97 0.98**	0.00 0.01 0.01
North	0.00 0.03 0.02	**1.00 0.99 0.99**

Table S4D. Power to distinguish among *SW vs SC vs SE*.

	SW	*SC*	*SE*
	Pr Rrej Rnn	*Pr Rrej Rnn*	*Pr Rrej Rnn*
SW	**0.53 0.48 0.46**	0.46 0.48 0.45	0.02 0.04 0.07
SC	0.44 0.46 0.46	**0.51 0.44 0.46**	0.03 0.04 0.08
SE	0.03 0.06 0.08	0.03 0.08 0.09	**0.95 0.92 0.85**

Table S4E. Power to distinguish among *SW vs SE*.

	SW	*SE*
	Pr Rrej Rnn	*Pr Rrej Rnn*
SW	**0.94 0.93 0.88**	0.02 0.05 0.09
SE	0.06 0.07 0.12	**0.98 0.95 0.91**

Table S5. Power of the ABC method for the parameters estimation. For every parameter under study, the table shows boxplots with the distance between the true and estimated (median, mean and mode) parameter value. This analysis was performed with the 100 *test datasets* (*LDD* scenario) and for all the studied parameters (T_{ANC}, N_{ANC}, N_{GR}, MIG_R, K, LDD_P and MUT_R). As a reference, the dashed line indicates that the simulated (true) and estimated values are equal (error = 0). The estimations were performed with the multiple regression adjustment implemented in *ABCtoolBox*[35].

	Error of the estimation		
**Parameter**	**Mode**	**Mean**	**Median**
Time of the onset of the expansion (T_{ANC})	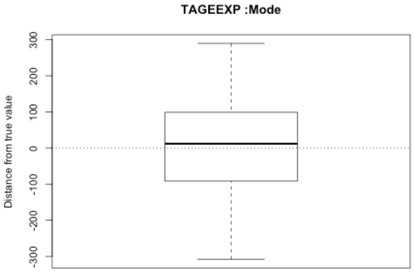	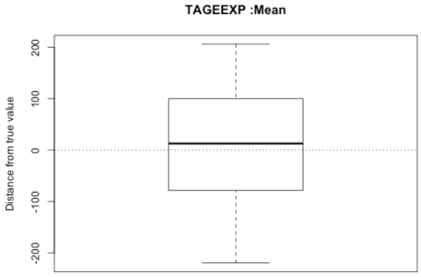	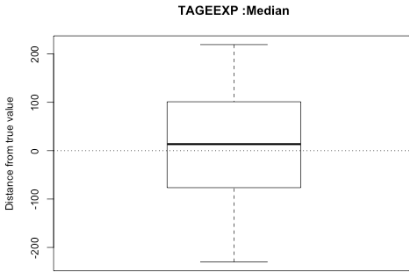
Population size at the onset of the expansion (N_{ANC})	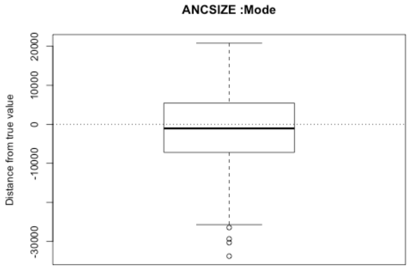	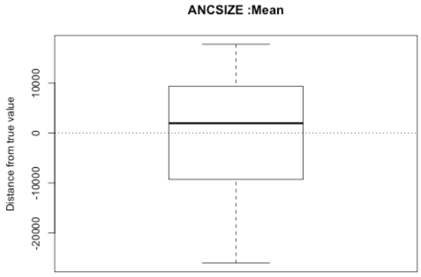	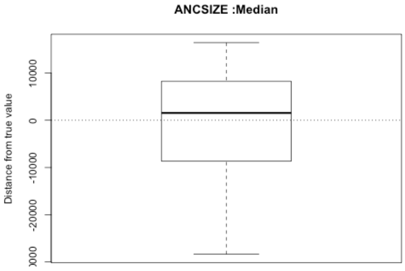
Population growth rate (N_{GR})	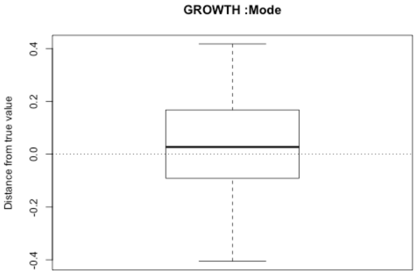	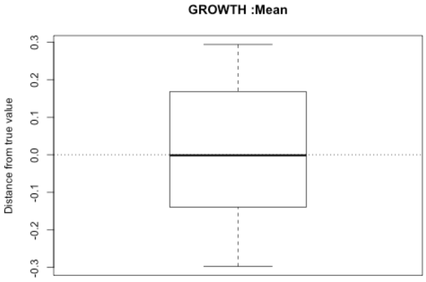	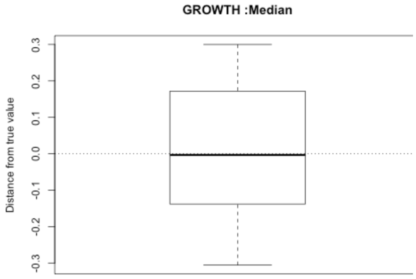
Migration rate (MIG_R)	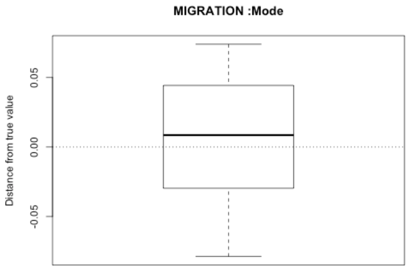	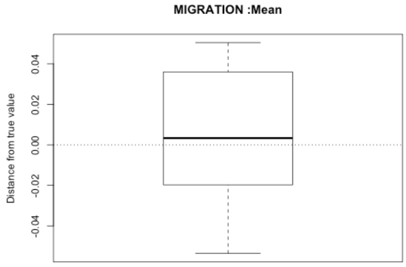	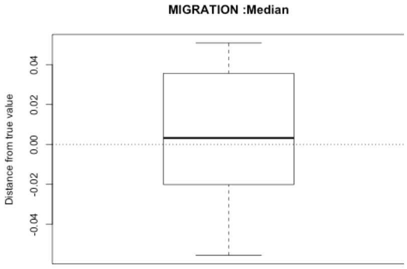

Carrying capacity (K)

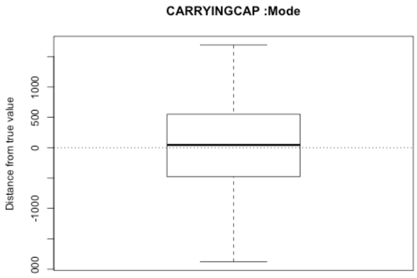

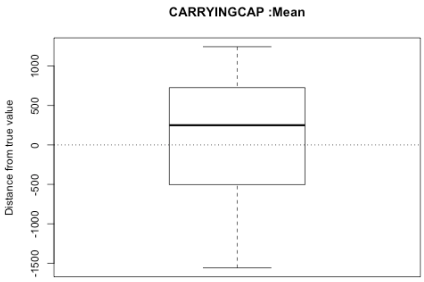

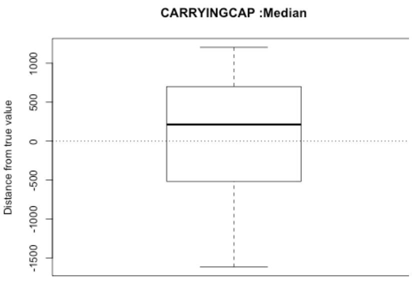

Proportion of LDD events (LDD_P)

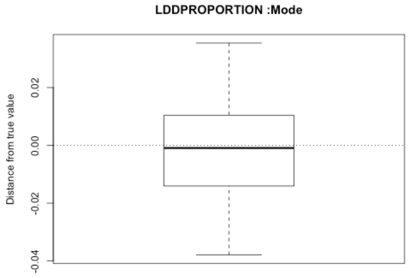

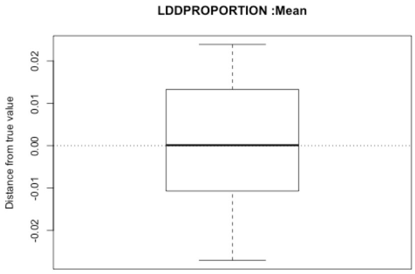

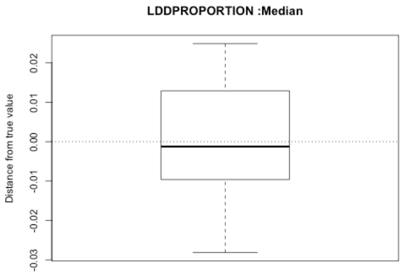

Mutation rate (MUT_R)

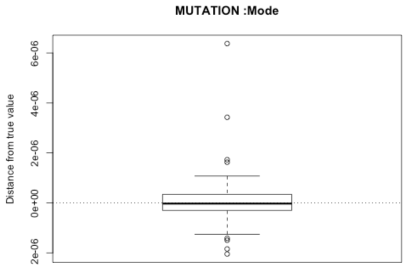

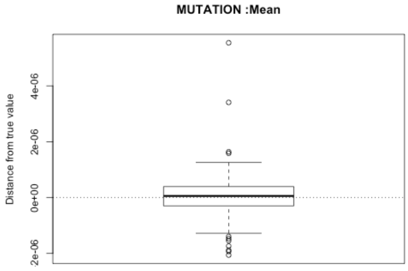

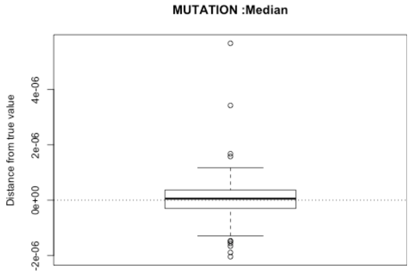

Supplementary figures

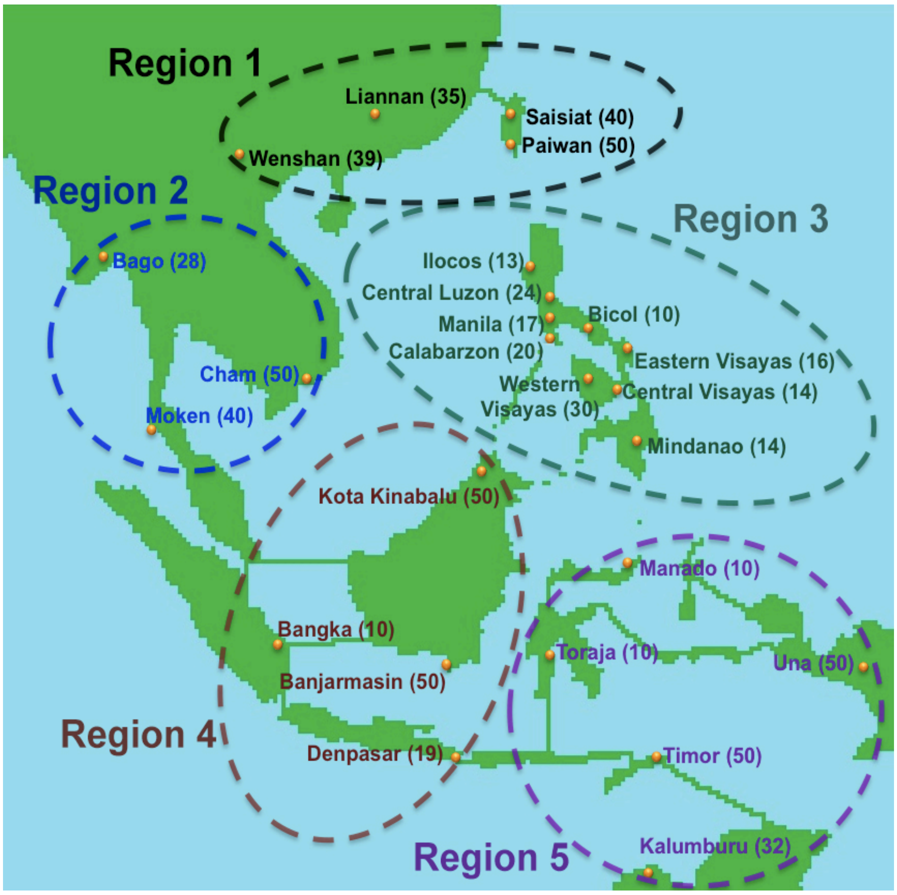

Figure S1. Sample locations, sample sizes and groups of geographic regions considered for the design of the summary statistics. Sample sizes are shown in parenthesis. Group 1 includes samples from the northern part of the continent of this landscape, Group 2 includes samples from the northern part of the continent of this landscape, Group 3 includes samples from the Philippines, Groups 4 and 5 involve the remaining samples separated according to the Wallace line. Note that for the computer simulations some artificial corridors were included to allow the colonization of diverse islands by connecting nearby small islands.

A

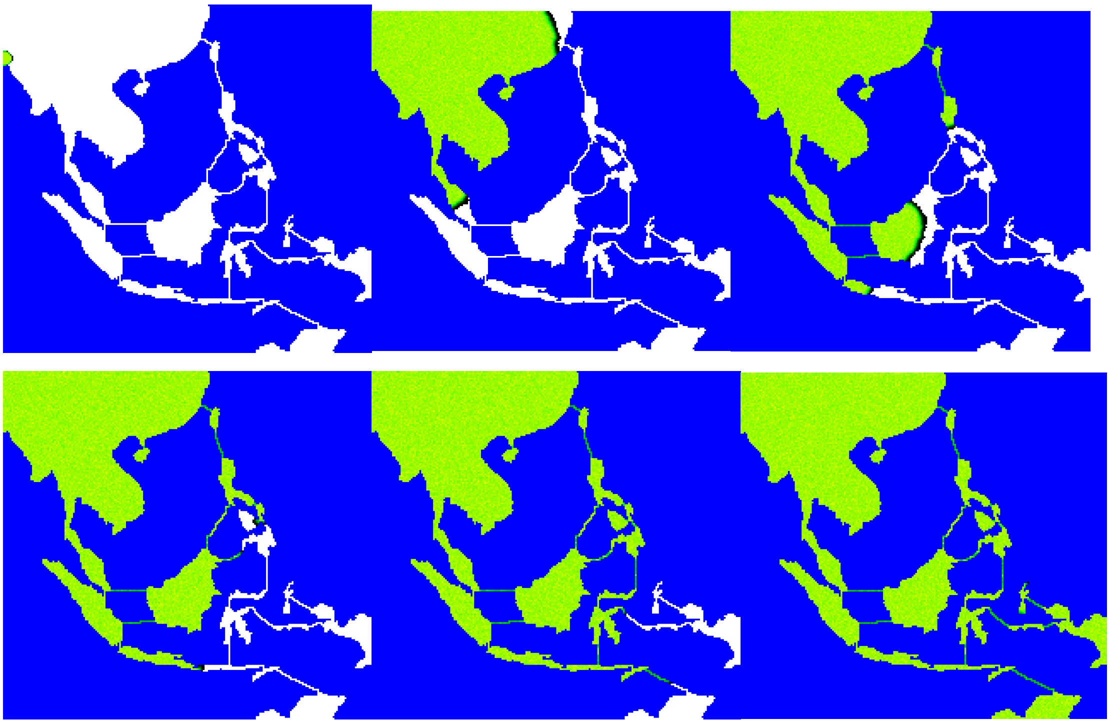

B

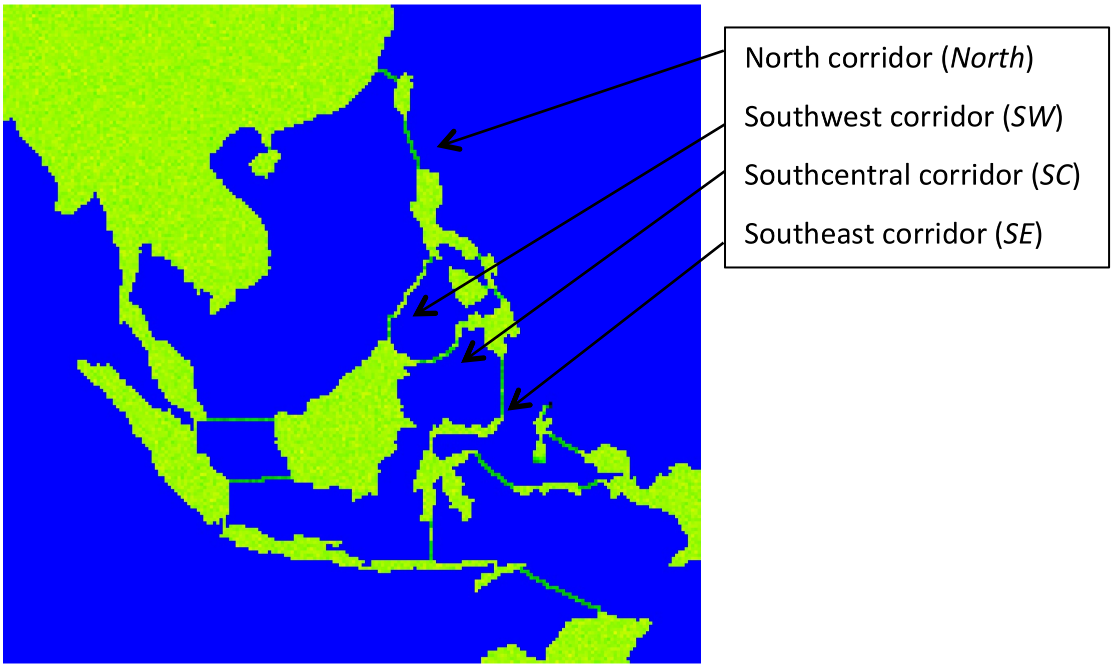
North corridor (*North*)
Southwest corridor (*SW*)
Southcentral corridor (*SC*)
Southeast corridor (*SE*)

C

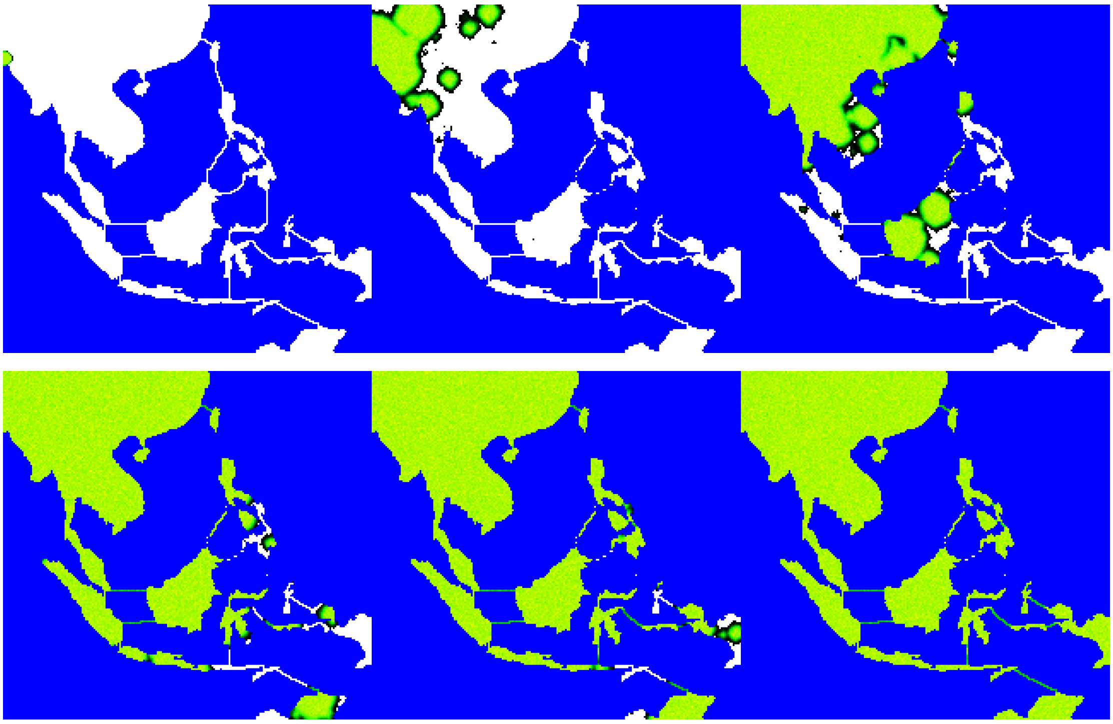

Figure S2. Illustrative examples of spatially explicit computer simulations to colonize the Philippines. Parameters to obtain these illustrative snapshots were randomly chosen within the prior distributions of the population genetics parameters (Table S2). A) Colonization through the North route and the three South routes (*AllCorr*) at generations 0, 300, 500, 600, 800 and 1,100 (from left to right). B) Migration routes used in scenarios without LDD. C) Colonization under LDD (*LDD*) at generations 0, 75, 125, 175, 200 and 275 (from left to right). Note that the landscape is colonized much faster with the presence of LDD events.

Figures S3. Fitting of the non-nested evolutionary scenarios of migration with the real data. The posterior probabilities of evolutionary scenarios were estimated with three different ABC methods: the Pritchard's method[32] (*Pr*) under a tolerance of 0.1% and the rejection (*Rrej*) and neuralnet (*Rnn*) methods (under a tolerance of 1% according to the author's recommendation) implemented in the *abc* package[33]. Figures S3A-E show the probabilities of the three ABC methods when fitting the studied different combinations of non-nested evolutionary scenarios with the real data.

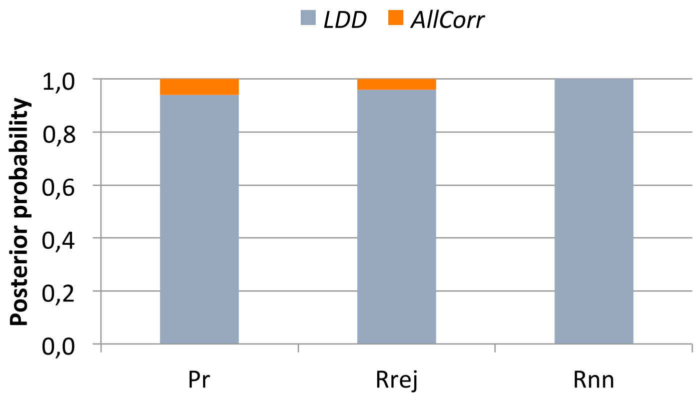

Figure S3A. Fitting of the evolutionary scenarios of migration routes (*LDD* and *AllCorr*) with the real data. Note that the LDD scenario fits better the real data.

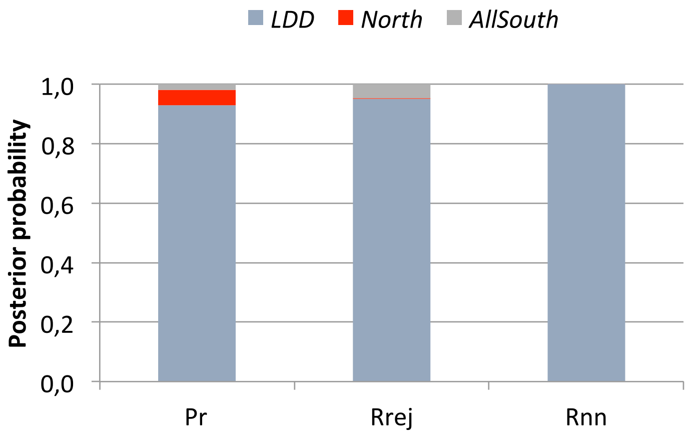

Figure S3B. Fitting of the evolutionary scenarios of migration routes (*LDD, AllSouth* and *North*) with the real data. Note that the LDD scenario fits better the real data.

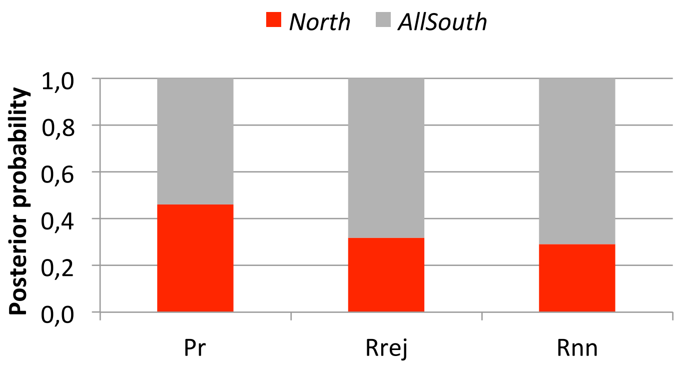

Figure S3C. Fitting of the evolutionary scenarios of migration routes (AllSouth and *North*) with the real data. Note that both scenarios do not present very distant probabilities of fitting with the real data, hence both scenarios could have occurred.

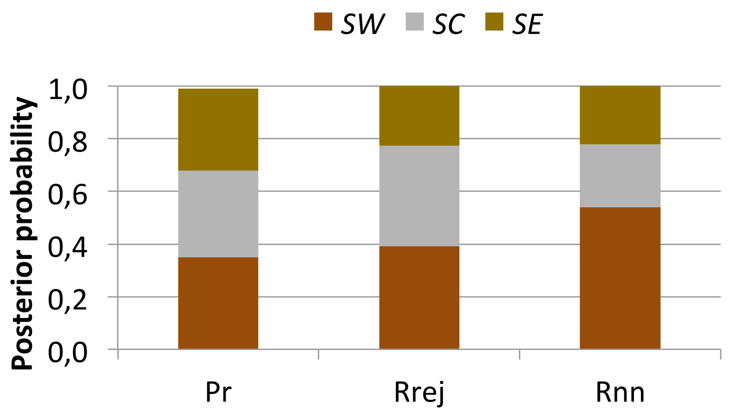

Figure S3D. Fitting of the evolutionary scenarios of migration routes (*SW*, *SC* and *SE*) with the real data. Note that the three scenarios do not present very distant probabilities of fitting with the real data, hence all of them could have occurred.

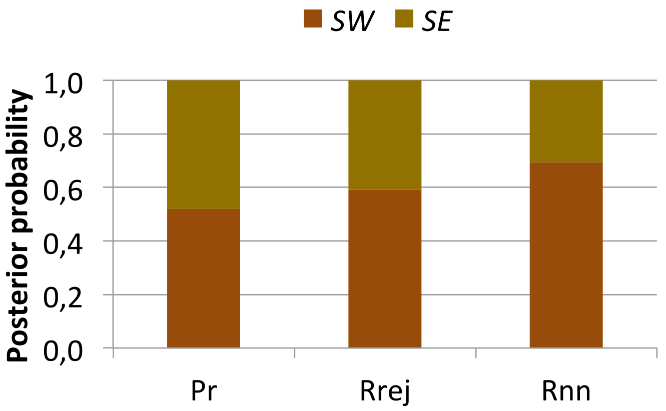

Figure S3E. Fitting of the evolutionary scenarios of migration routes (*SW* and *SE*) with the real data. Note that both scenarios do not present very distant probabilities of fitting with the real data, hence both scenarios could have occurred.

Figure S4. Goodness of fit for the evaluation of the evolutionary scenarios with the real data. Although the goodness of fit is usually applied to only the best fitting scenario before turning to parameter inference and in order to check that the preferred scenario provides a good fitting to the real data[33] here we analyzed the goodness of fit in all the investigated scenarios to show an additional evaluation of the fitting with the real data, but it is expected that less fitting scenarios also generate a poorer goodness of fit. Above: A principal component analysis (PCA) with two components is applied to make new summary statistics. The plot displays the 90% envelope (following the settings and recommendations indicated in the documentation of the package *abc*[33]) of the two PCs obtained from the SS of data simulated under each evolutionary scenario. The cross represents the SS from the real data. Note that the scenario that better fits the real data always presents the cross (real data) more centered into its surface, in other words, the SS of the real data are closer to the SS of the simulated data under such a scenario. Below: Evaluation of distance between SS from real data (blue vertical line) and null distributions of simulated SS of every scenario. Note that, the goodness of fit is in agreement with the evaluation of the selection among scenarios (Figure S3) by selecting the best fitting scenario.

LDD vs AllCorr

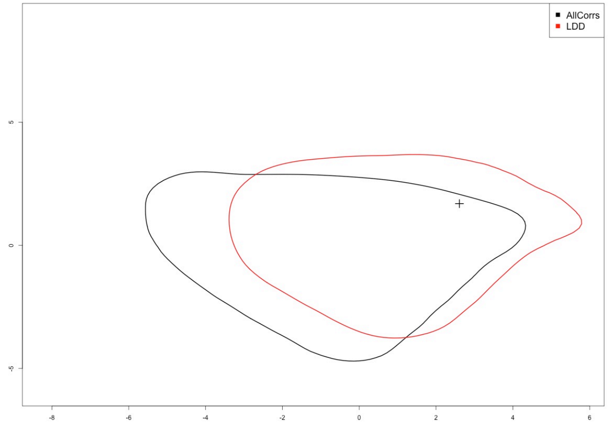

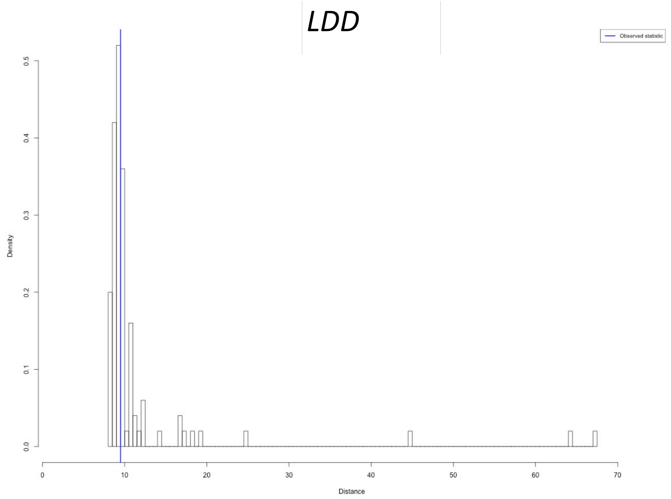

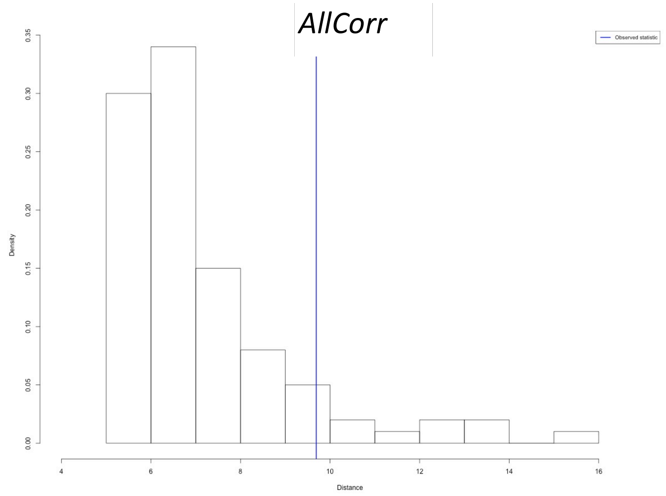

LDD vs AllSouth vs North

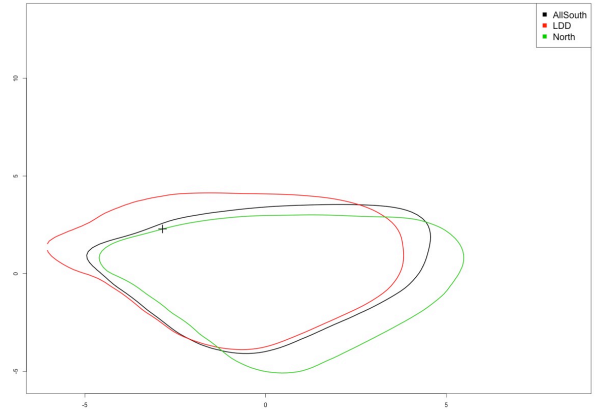

LDD

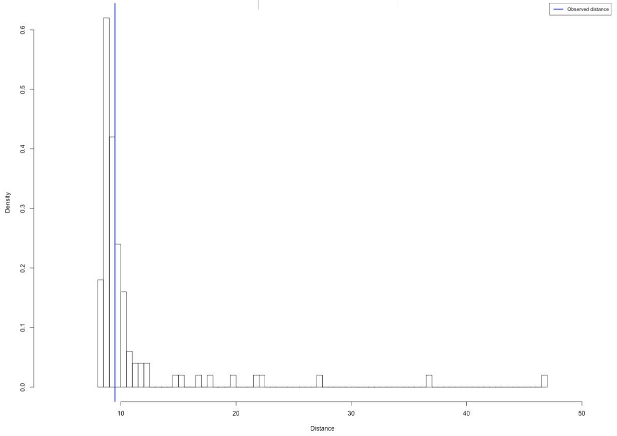

AllSouth

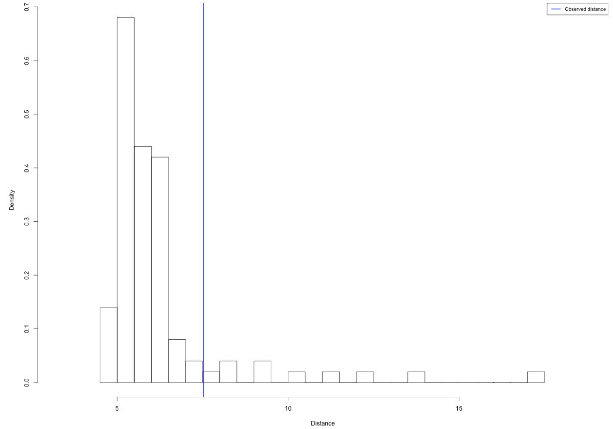

North

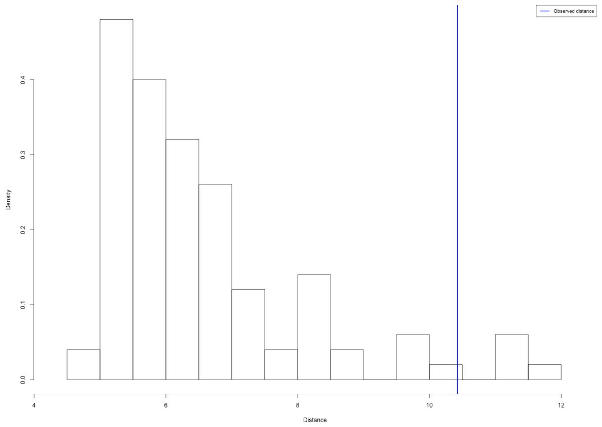

AllSouth vs North

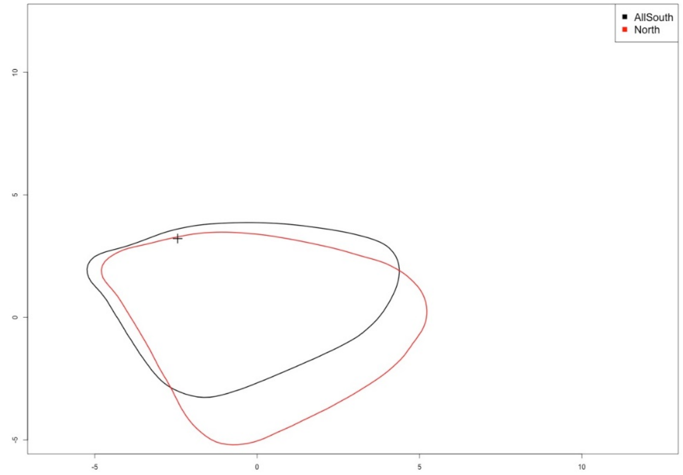

AllSouth

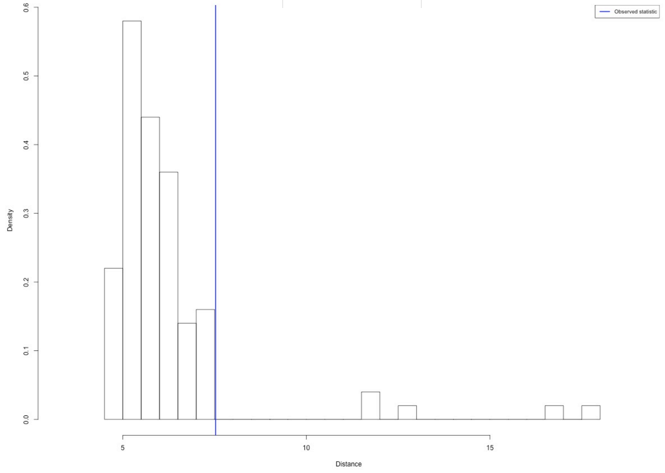

North

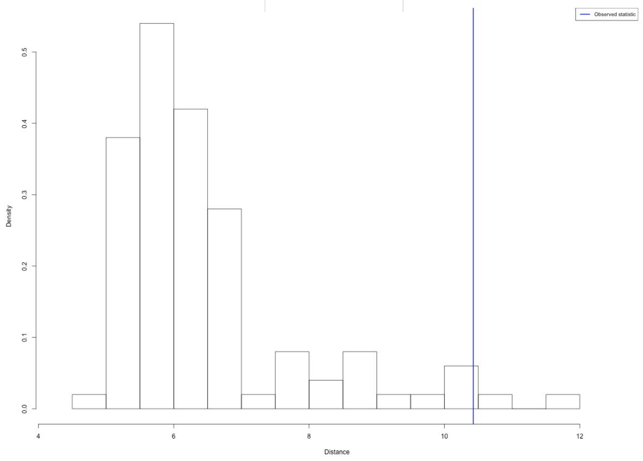

SW vs SC vs SE

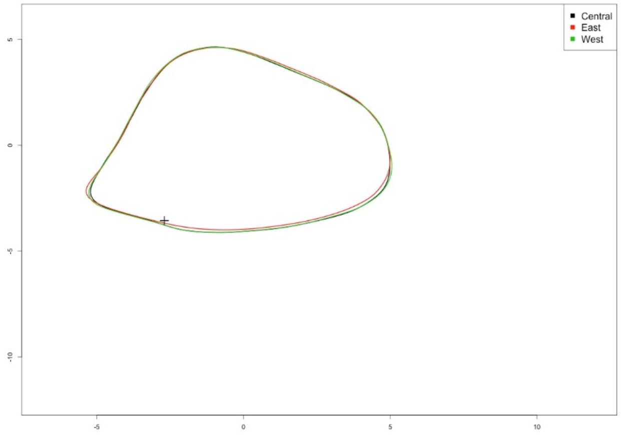
Central
East
West

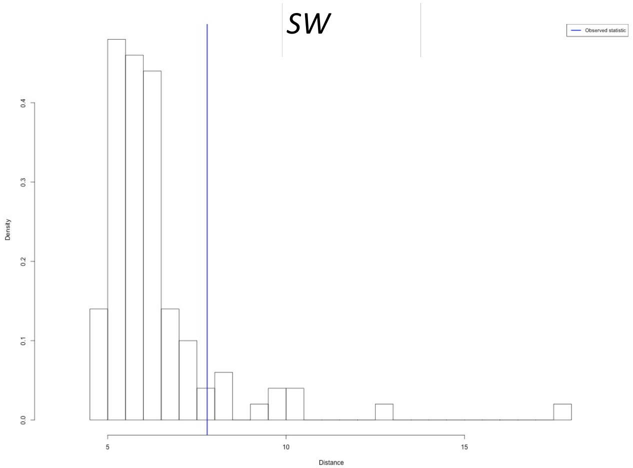
SW
Observed statistic
Density
Distance

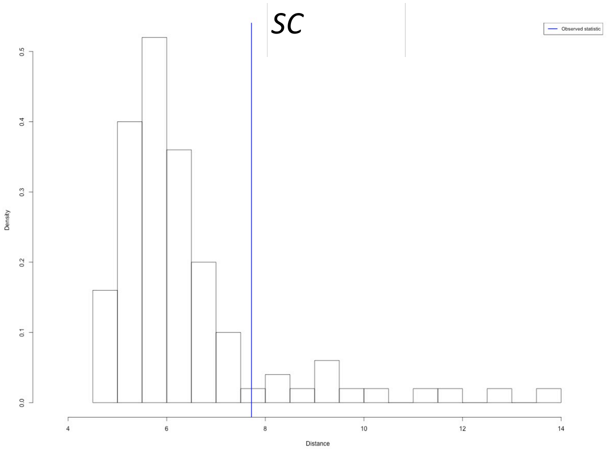
SC
Observed statistic
Density
Distance

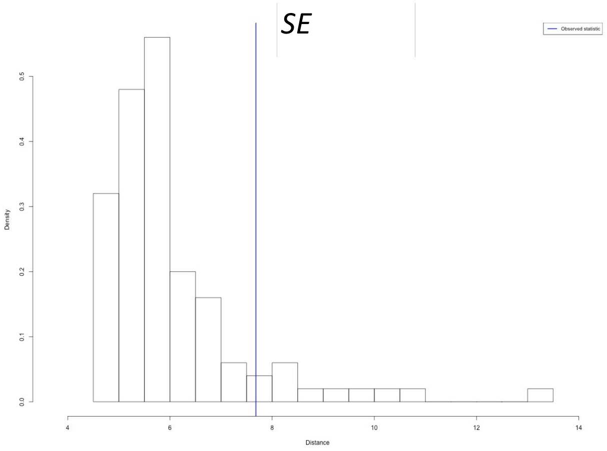
SE
Observed statistic
Density
Distance

SW vs SE

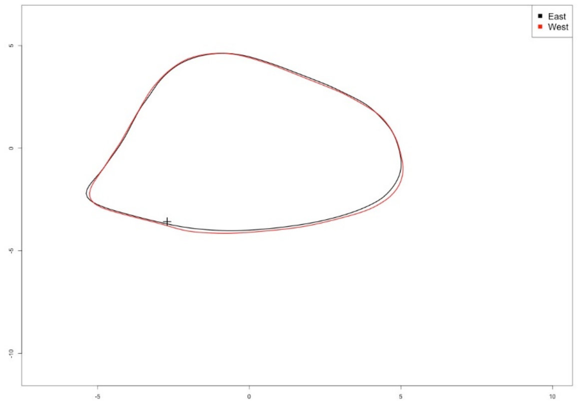
East
West

SW

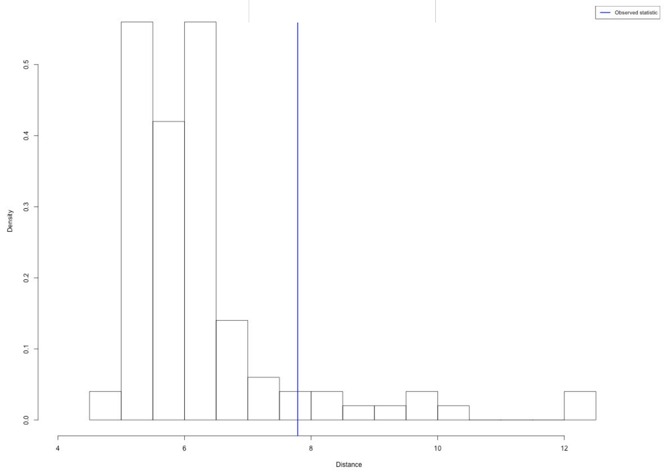
Observed statistic
Density
Distance

SE

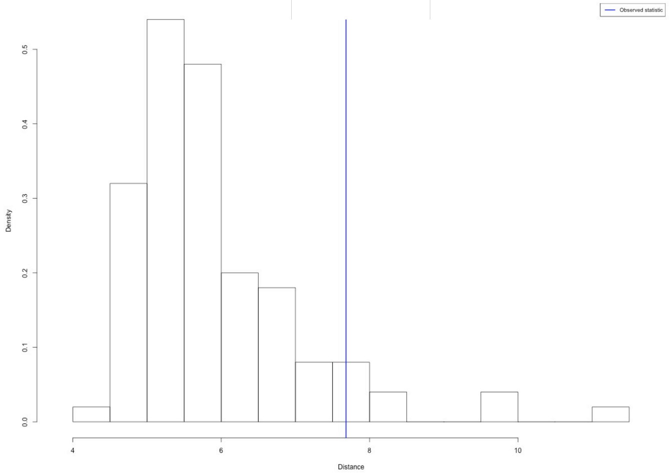
Observed statistic
Density
Distance

References cited in the Supplementary Material

1 Currat, M., Arenas, M., Quilodran, C. S., Excoffier, L. & Ray, N. SPLATCHE3: simulation of serial genetic data under spatially explicit evolutionary scenarios including long-distance dispersal. *Bioinformatics* **In press** (2019).

2 Arenas, M., Francois, O., Currat, M., Ray, N. & Excoffier, L. Influence of admixture and paleolithic range contractions on current European diversity gradients. *Mol Biol Evol* **30**, 57-61 (2013).

3 Benguigui, M. & Arenas, M. Spatial and temporal simulation of human evolution. Methods, frameworks and applications. *Curr Genomics* **15**, 245-255 (2014).

4 Currat, M., Poloni, E. S. & Sanchez-Mazas, A. Human genetic differentiation across the Strait of Gibraltar. *BMC Evol Biol* **10**, 237 (2010).

5 Currat, M. & Excoffier, L. The effect of the Neolithic expansion on European molecular diversity. *Proc Biol Sci* **272**, 679-688 (2005).

6 Pimenta, J., Lopes, A. M., Comas, D., Amorim, A. & Arenas, M. Evaluating the Neolithic Expansion at Both Shores of the Mediterranean Sea. *Mol Biol Evol* **34**, 3232-3242 (2017).

7 Branco, C., Velasco, M., Benguigui, M., Currat, M., Ray, N. & Arenas, M. Consequences of diverse evolutionary processes on american genetic gradients of modern humans. *Heredity*, in press (2018).

8 Currat, M. & Excoffier, L. Modern Humans Did Not Admix with Neanderthals during Their Range Expansion into Europe. *PLoS Biol* **2**, e421 (2004).

9 Kimura, M. & Weiss, G. H. The Stepping Stone Model of Population Structure and the Decrease of Genetic Correlation with Distance. *Genetics* **49**, 561-576 (1964).

10 Ray, N. & Excoffier, L. A first step towards inferring levels of long-distance dispersal during past expansions. *Mol Ecol Resour* **10**, 902-914 (2010).

11 Currat, M., Ray, N. & Excoffier, L. SPLATCHE: a program to simulate genetic diversity taking into account environmental heterogeneity. *Mol Ecol Notes* **4**, 139-142 (2004).

12 Yang, Z. *Computational Molecular Evolution*. (Oxford University Press, 2006).

13 Arenas, M. Simulation of Molecular Data under Diverse Evolutionary Scenarios. *PLoS Comput Biol* **8**, e1002495 (2012).

14 Excoffier, L., Foll, M. & Petit, R. J. Genetic consequences of range expansions. *Annu Rev Ecol Evol Syst* **40**, 481–501 (2009).

15 Wen, B. *et al.* Genetic structure of Hmong-Mien speaking populations in East Asia as revealed by mtDNA lineages. *Mol Biol Evol* **22**, 725-734 (2005).

16 Tajima, A., Sun, C. S., Pan, I. H., Ishida, T., Saitou, N. & Horai, S. Mitochondrial DNA polymorphisms in nine aboriginal groups of Taiwan: implications for the population history of aboriginal Taiwanese. *Hum Genet* **113**, 24-33 (2003).

17 Trejaut, J. A. *et al.* Traces of archaic mitochondrial lineages persist in Austronesian-speaking Formosan populations. *PLoS Biol* **3**, e247 (2005).

18 Summerer, M. *et al.* Large-scale mitochondrial DNA analysis in Southeast Asia reveals evolutionary effects of cultural isolation in the multi-ethnic population of Myanmar. *BMC Evol Biol* **14**, 17 (2014).

19 Pradutkanchana, S., Ishida, T. & Kimura, R. Mitochondrial diversity of the sea nomads of Thailand. *Unpublisheb* (2010).
20 Peng, M. S. *et al.* Tracing the Austronesian footprint in Mainland Southeast Asia: a perspective from mitochondrial DNA. *Mol Biol Evol* **27**, 2417-2430 (2010).
21 Hill, C. *et al.* A mitochondrial stratigraphy for island southeast Asia. *Am J Hum Genet* **80**, 29-43 (2007).
22 Hill, C. *et al.* Phylogeography and ethnogenesis of aboriginal Southeast Asians. *Mol Biol Evol* **23**, 2480-2491 (2006).
23 Macaulay, V. *et al.* Single, rapid coastal settlement of Asia revealed by analysis of complete mitochondrial genomes. *Science* **308**, 1034-1036 (2005).
24 Hudjashov, G. *et al.* Revealing the prehistoric settlement of Australia by Y chromosome and mtDNA analysis. *Proc Natl Acad Sci U S A* **104**, 8726-8730 (2007).
25 Tommaseo-Ponzetta, M., Attimonelli, M., De Robertis, M., Tanzariello, F. & Saccone, C. Mitochondrial DNA variability of West New Guinea populations. *Am J Phys Anthropol* **117**, 49-67 (2002).
26 Gomes, S. M. *et al.* Human settlement history between Sunda and Sahul: a focus on East Timor (Timor-Leste) and the Pleistocenic mtDNA diversity. *BMC Genomics* **16**, 70 (2015).
27 Oppenheimer, S. Out-of-Africa, the peopling of continents and islands: tracing uniparental gene trees across the map. *Philos Trans R Soc Lond B Biol Sci* **367**, 770-784 (2012).
28 Tenesa, A., Navarro, P., Hayes, B. J., Duffy, D. L., Clarke, G. M., Goddard, M. E. & Visscher, P. M. Recent human effective population size estimated from linkage disequilibrium. *Genome Res* **17**, 520-526 (2007).
29 Soares, P. *et al.* Correcting for purifying selection: an improved human mitochondrial molecular clock. *Am J Hum Genet* **84**, 740-759 (2009).
30 Henn, B. M., Gignoux, C. R., Feldman, M. W. & Mountain, J. L. Characterizing the time dependency of human mitochondrial DNA mutation rate estimates. *Mol Biol Evol* **26**, 217-230 (2009).
31 Madrigal, L. *et al.* High mitochondrial mutation rates estimated from deep-rooting costa rican pedigrees. *Am J Phys Anthropol* **148**, 327-333 (2012).
32 Pritchard, J. K., Seielstad, M. T., Perez-Lezaun, A. & Feldman, M. W. Population growth of human Y chromosomes: a study of Y chromosome microsatellites. *Mol Biol Evol* **16**, 1791-1798 (1999).
33 Csillery, K., Francois, O. & Blum, M. G. B. abc: an R package for approximate Bayesian computation (ABC). *Methods in Ecology and Evolution* **3**, 475-479 (2012).
34 Beaumont, M. A., Zhang, W. & Balding, D. J. Approximate Bayesian computation in population genetics. *Genetics* **162**, 2025-2035 (2002).
35 Wegmann, D., Leuenberger, C., Neuenschwander, S. & Excoffier, L. ABCtoolbox: a versatile toolkit for approximate Bayesian computations. *BMC Bioinformatics* **11**, 116 (2010).
